# Novel molecular design via a scaffold-aware transformer with multi-scale attention mechanisms

**DOI:** 10.1186/s13321-026-01221-6

**Published:** 2026-05-19

**Authors:** Junyoung Park, Sunyong Yoo

**Affiliations:** 1 Department of Intelligent Electronics and Computer Engineering, Chonnam National University, Gwangju, 61186 Republic of Korea; 2 R&D Center, MATILO AI Inc., Gwangju, 61186 Republic of Korea

**Keywords:** Generative model, De novo molecular design, Drug discovery, Attention, Transformer

## Abstract

Recent advancements in artificial intelligence have demonstrated enhanced potential in accelerating drug discovery by exploring vast chemical spaces and predicting molecular properties. In particular, scaffold-constrained molecular generation has enabled the incorporation of structural constraints into the generation process. However, effectively integrating multi-scale structural information with activity-guided optimization remains challenging. To overcome these limitations, we propose a novel framework that integrates a transformer-based generative model and a graph attention network-based predictive model. The generative model produces molecules with desired structural characteristics by explicitly incorporating scaffold information, while the predictive model estimates the biological activity of the generated molecules. A supervised fine-tuning framework iteratively refines the generator through multi-stage tournament selection with experience memory. This framework guides the generator toward scaffold-consistent, high-affinity candidates while exploring novel chemical variations around a user-specified scaffold. Experimental results demonstrated that the proposed scaffold-aware transformer achieves competitive validity, uniqueness, and novelty, effectively generating novel compounds with high predicted binding affinity for biological targets. Meanwhile, an attention-based analysis extracted atom-level importance scores, highlighting the substructures that contribute to the predicted binding affinity and providing interpretable insights into structure–activity relationships. This study provides a practical and interpretable tool for scaffold-conditioned molecular generation.

**Supplementary Information:**

The online version contains supplementary material available at 10.1186/s13321-026-01221-6.

## Scientific contribution

This study introduces a scaffold-conditioned generative framework that enables explicit structural control by incorporating user-specified core molecular frameworks into the generation process. A multi-scale attention mechanism captures scaffold features at multiple resolutions, simultaneously learning local atom-bond neighborhoods and global scaffold topology. Additionally, attention-based analysis provides interpretable insights into structure–activity relationships by highlighting substructures contributing to predicted binding affinity.

**Supplementary Information:**

The online version contains supplementary material available at 10.1186/s13321-026-01221-6.

## Introduction

Drug development is a complex and resource-intensive process that faces multiple interconnected challenges. The conventional drug development pipeline takes 10–15 years from concept to approval and costs billions of dollars [1]. In particular, designing molecular structures in the early stages of development is a challenging task owing to the vast chemical search space for new molecules [2]. The number of potential drug-like molecules is estimated to be around ${10}^{60}$, yet only about ${10}^{8}$ molecules have been synthesized to date [3]. Furthermore, despite extensive screening efforts in which thousands of candidate compounds are synthesized, only a small fraction possess sufficient biological activity and safety to advance into clinical trials [4]. The challenges persist even at later stages, with approximately 90% of drug candidates that enter clinical testing ultimately failing due to insufficient efficacy, toxicity, or a lack of drug-like properties [5]. Thus, these issues underscore the urgent need for diversification in drugs and improved discovery strategies [6–9].

Artificial intelligence is emerging as a means to overcome the limitations in molecular design by accelerating the exploration of the vast chemical space, thereby reducing the time and cost required to derive candidate substances [1, 2, 10–13]. De novo molecular design is a computational paradigm for generating novel chemical structures with desired properties. Recently, generative models have been actively pursued in drug discovery, with several studies demonstrating experimental validation of computationally designed molecules [14–18]. These approaches leverage generative models to learn the distribution of molecules with target-specific activity and to sample novel chemical structures [17]. These models promise to discover candidate substances much faster and more efficiently than traditional synthetic chemistry methodologies. Many of these approaches utilize the Simplified Molecular Input Line Entry System (SMILES), a text-based representation that encodes molecular structures as strings, thereby enabling the application of natural language processing techniques in chemistry [19]. Generative models trained on SMILES can learn chemical syntax and generate syntactically valid molecules, with some frameworks incorporating reinforcement learning or evolutionary algorithms to optimize the generated compounds [20, 21]. Several approaches have been developed to incorporate structural constraints into molecular generation, including scaffold-constrained methods such as LibINVENT and fragment-based approaches such as MoLeR [18, 22, 23]. While these methods enable scaffold-aware generation, integrating multi-scale structural information with iterative activity-guided optimization presents additional opportunities for improvement. Separately, LOGICS proposed a framework utilizing tournament selection and experience replay for iterative bioactivity optimization [15]. By integrating generative and predictive models, this approach enables property prediction during generation and provides a method to learn optimized distributions of bioactive molecules. However, this approach still has the limitation that it cannot explicitly control the scaffold structure. In medicinal chemistry, the scaffold is the core framework that defines molecular topology and guides key substituent vectors. Early selection and explicit control of the scaffold are central to steering potency, selectivity, and developability, because scaffold changes are labor-intensive and often erode activity [24, 25]. Without such scaffold control, the generated molecules may lack the structural characteristics necessary for lead quality.

To address these limitations, this study proposes a scaffold-aware generative framework that integrates structural control with continuous optimization of bioactivity. Our approach combines a transformer-based generative model and a graph attention network (GAT)-based predictive model within a supervised fine-tuning (SFT) framework [15, 26, 27]. We integrate scaffold information into the generation process through multi-scale attention, which enables the simultaneous control of local atom-bond neighborhoods and global scaffold topology. The GAT-based predictive model estimates the biological activity of generated molecules. Through an iterative interaction between the generator and predictor, the framework continuously evaluates and refines molecules throughout the training process. We further employ a tournament-based selection mechanism with experience memory to guide subsequent learning iterations. This framework enables the exploration of new variations while maintaining the core structure of the molecule.

## Materials and methods

### Datasets

This study utilized two distinct types of datasets: a molecular design benchmark dataset for training the generative model and a biological activity-labeled dataset for training the predictive model. The statistical characteristics of the datasets are summarized in Table 1.

**Table 1:** Statistical overview of the datasets utilized in this study

Datasets	Model	Train	Validation	Test	Total
GuacaMol	Generator	1,260,532	78,762	236,374	1,575,668
KOR	Predictor	2,674	573	574	3,821
PIK3CA	Predictor	1,023	219	220	1,462

The molecular design benchmark dataset, GuacaMol, was utilized to train the generative model [28]. GuacaMol comprises 1,591,378 molecules extracted from the ChEMBL database [29]. Data preprocessing for training the generator was performed as follows. First, SMILES strings were standardized and deduplicated to enhance the training efficiency of the generative model. Second, Bemis–Murcko scaffolds were extracted from each SMILES string to identify the core structural framework of the molecules [30]. RDKit was used in both steps [31]. To ensure structural consistency and relevance, we removed molecules from the dataset for which scaffolds could not be extracted—typically those with simple linear structures or those lacking a basic framework. Third, we defined a vocabulary to facilitate the tokenization of the SMILES in the model [32]. Fourth, we defined and applied a regular expression pattern to tokenize the SMILES strings into meaningful units [33]. Since the SMILES includes a wide range of chemical symbols and bond representations, precise tokenization is crucial for the model to learn molecular structural information. Fifth, special tokens—[SOS] (start of sequence) and [EOS] (end of sequence)—were appended to each tokenized SMILES string to denote the beginning and end of each molecular sequence. Finally, padding tokens were added to ensure that all SMILES strings within the dataset had uniform lengths. This uniformity was necessary to ensure efficient batch processing during the model training, allowing the generator to handle sequences of consistent dimensions.

We used biological activity datasets for the KOR (κ-opioid receptor) and PIK3CA (phosphatidylinositol 3-kinase catalytic subunit alpha) proteins. Both datasets were sourced from preprocessed sets provided by a previous study [15]. The original preprocessing pipeline consisted of canonicalization of the SMILES and the removal of tokens not present in the vocabulary; for example, tokens such as '[Br-]', '[I-]', and '[Cl-]' were excluded. Additionally, when a compound had multiple bioactivity measurements, the median value was chosen as its representative label. The bioactivity values for the KOR were supplied as the pIC_50_. Meanwhile, the bioactivity values for PIK3CA were provided as pKi and pKd—metrics that represent the negative logarithms of the inhibition (Ki) and dissociation (Kd) constants [34–36]. To enable unified activity prediction, the pKi and pKd values were merged into a single measure, pKx. Activity thresholds were established to distinguish between active and inactive compounds. For KOR, molecules with a pIC_50_ value of 7.0 or higher were classified as active, while molecules with a pKx value of 8.0 or higher were classified as active for PIK3CA [15].

### Framework architecture

**Fig. 1:**
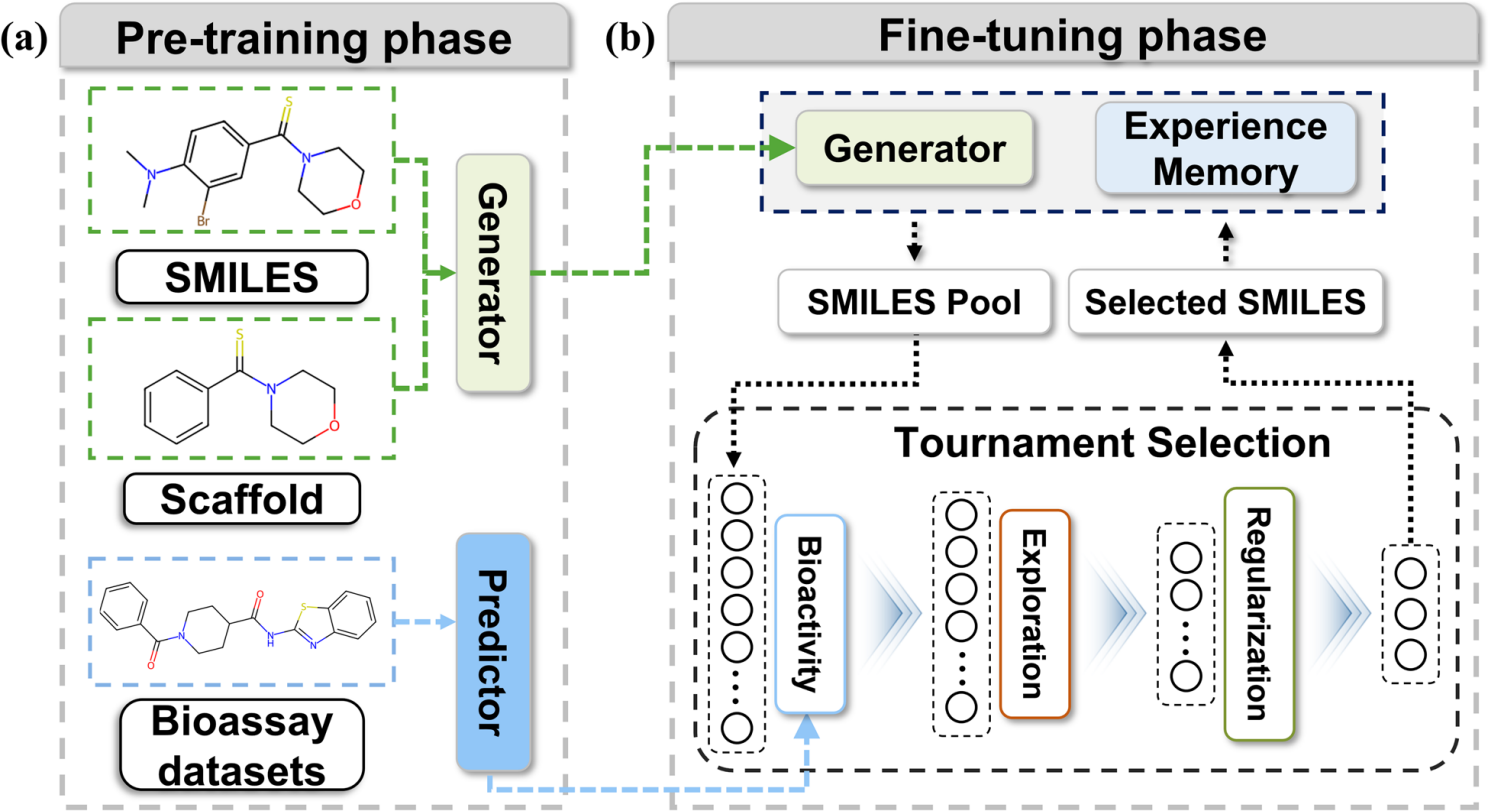
Overall training framework with pre-training and fine-tuning. **a** Pre-training phase. The generator is trained on the SMILES and the corresponding scaffolds, while the predictor is trained on bioassay datasets. **b** Fine-tuning phase. The generator samples candidate SMILES, and the experience memory extracts previously stored SMILES; these were combined to form a SMILES pool. A multi-stage tournament selection is then applied to the pool to obtain the selected SMILES, which are used to retrain the generator and to update the experience memory

An overview of the framework architecture is presented in Fig. 1. The framework consists of two phases: pre-training and SFT. During pre-training, the generator learns to produce SMILES conditioned on input scaffolds, while the predictor is independently trained to estimate binding affinities. During the SFT phase, the generator and predictor interact iteratively: the generator produces candidate molecules, the predictor evaluates their binding affinities, and high-affinity molecules are selected through tournament selection to update the generator.

### Generative model

**Fig. 2:**
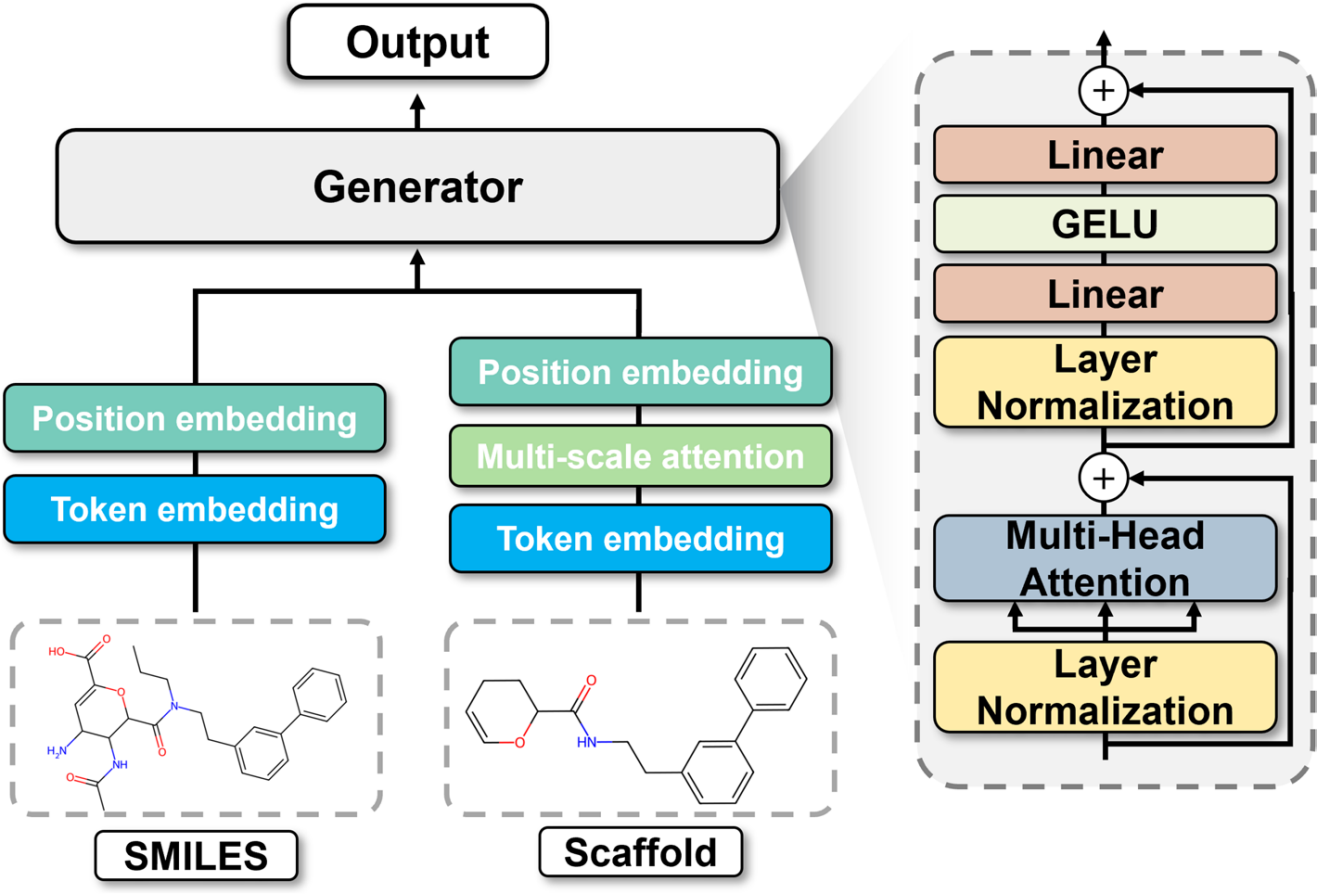
The architecture of the generative model. Input SMILES and scaffolds are converted into numerical representations through token embedding. For scaffolds, multi-scale attention extracts structural features at different scales. Both inputs are combined with positional embeddings to preserve sequential information before being fed into the generator. The generator comprises multiple decoder blocks, each containing layer normalization, multi-head attention, and a feed-forward network with residual connections. Output is sampled from the generator

The generative model is based on the transformer architecture, which utilizes a self-attention mechanism to process sequential data (Fig. 2). Conventional sequence models such as RNN and LSTM process tokens sequentially, which causes gradient signals to diminish over long sequences and makes it difficult to capture dependencies between distant tokens. The transformer overcomes these limitations through its self-attention mechanism, which computes direct pairwise relationships between all positions in the sequence regardless of their distance, enabling effective modeling of long-range dependencies while allowing parallel computation [26, 37, 38]. This can effectively address long-term dependency issues. The core of the transformer is its attention mechanism, which learns how various positions in the input sequence relate to one another, allowing the model to focus on important information. Each input token is converted into three vectors: query, key, and value. The attention score is derived by first calculating the dot product of the query and key vectors, then normalizing the result using the softmax function. The final representation for each token is obtained by multiplying the normalized attention scores by the value vectors. The following formula can be employed to represent this process:

1 $$A\left(Q,K,V\right)= softmax\left(Q{K}^{T}\right)V$$

In Eq. 1, A(Q,K,V) is the attention value, Q is the query vector, K is the key vector, and V is the value vector. This mechanism operates in parallel across multiple attention heads.

The input SMILES and scaffolds are converted into token embeddings. For the scaffold inputs, we introduced a multi-scale attention mechanism to effectively generate molecules with desired structural characteristics [39]. This mechanism extracts structural information from the scaffold at various scales and incorporates it into the model. For example, when the scale is 1, the original scaffold embedding is used; when the scale is 2, grid sampling is applied to reduce its length by half; when the scale is 4, the length is reduced one-quarter. In this study, scales of 1, 2, and 4 were used to consider both local and global patterns of the scaffold. The attention outputs at each scale are linearly interpolated back to the original scaffold embedding length. Subsequently, the mean of the attention outputs is computed to provide an integrated representation of scaffold attention. This process can be formulated as follows:

2 $${X}_{s}=Downsample({X}_{scaffold}, s)$$

3 $$Z=\frac{1}{3}{\sum}_{s\in \left\{\mathrm{1,2},4\right\}}Interpolate(MH{A}_{s}\left({X}_{s}\right),L)$$

In Eqs. 2 and 3, ${X}_{scaffold}$ represents the scaffold token embeddings, s denotes the scale factor, ${X}_{s}$ is the downsampled scaffold embedding at scale s, $MH{A}_{s}$ is the multi-head attention operation at scale s, and L is the original scaffold sequence length. Positional encoding is then employed to provide the model with information about the order of the sequence**.** The token embeddings and positional embeddings are combined and used as inputs for the generator.

The generator consists of multiple transformer decoder blocks. In each decoder block, the multi-head attention enables the learning of relationships between each position in the input sequence and other positions simultaneously across various representation spaces. Following the multi-head attention layer, a feed-forward neural network is applied, consisting of two linear transformations and a Gaussian error linear unit function placed in between [40]. Layer normalization is employed before each sub-layer to normalize the input features. Additionally, residual connections are incorporated around each sub-layer, which mitigates the vanishing gradient problem and improves information flow through the network. The focal loss is applied to reduce class imbalance and improve training efficiency [41]. It serves as a reweighted negative log likelihood objective to stabilize SMILES token learning under highly skewed token frequencies. In SMILES sequences, a small subset of tokens and patterns occur far more often than others, and standard cross entropy can be dominated by these frequent and easily predicted tokens. Focal loss reduces the contribution of well classified tokens and preserves gradient signal for harder and less frequent tokens, which helps mitigate repetitive generation during training without altering the generation procedure itself. The focal loss function is defined as follows:

4 $$FL({p}_{t}) = -\alpha \times {(1-{p}_{t})}^{\gamma } \times log({p}_{t})$$

In Eq. 4, ${p}_{t}$ is the predicted probability for the target token; α is the weighting factor that controls relative weighting across tokens, and γ is the focusing parameter. γ functions to reduce the weighting on well-classified samples and increase the weighting on misclassified samples. The complete generator hyperparameters and training settings are provided in Supplementary Information Section 1 and Table S1. The SMILES generation process is described in the Supplementary Information Section 2 and Fig. S1.

### Predictive model

**Fig. 3:**
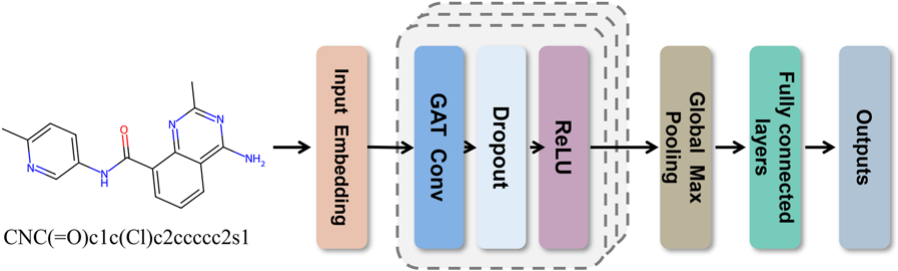
Workflow of the molecular prediction. The prediction process begins by converting input SMILES strings into molecular graphs. These graphs are passed through multiple GAT convolutional layers, which extract graph-level features. Features are processed via global max pooling and then fed into fully connected layers to predict the binding affinity

The predictive model comprises three GAT convolutional layers and fully connected layers (Fig. 3). The SMILES strings are converted into a graph form, where atoms serve as nodes and bonds as edges. Each node in the graph has a vector representing the features of that atom, such as atom type, charge state, and other physicochemical properties. These graph representations are then fed into the predictor. Each GAT convolutional layer learns interactions between nodes from multiple perspectives through a multi-head attention mechanism, effectively capturing complex features of the molecular structure. The core of GAT involves calculating attention coefficients that determine the importance of neighboring nodes when updating the feature vector of each node [27]. This is accomplished by first applying a linear transformation to each node feature vector and then computing attention scores between neighboring nodes. The attention scores are then normalized and used to weight the feature vectors of neighboring nodes, which are subsequently aggregated to update the feature vector of the node. The ReLU activation function is applied after each GAT layer. Additionally, dropout is used to prevent overfitting. Global max pooling is performed after the three GAT layers to extract a graph-level feature vector, which is then passed through fully connected layers to yield the final prediction.

During training, labeled SMILES data are used to predict the binding affinity between molecules and targets. Techniques such as weight decay and learning rate scheduling are applied during training. The mean squared error (MSE) is used as the loss function to formulate the task as a regression problem. The MSE quantitatively evaluates the prediction performance of the model by squaring and averaging the differences between the predicted and true activity values. The MSE is defined as follows:

5 $$MSE = \frac{1}{n} \sum_{i=1}^{n}{\left({y}_{i} - {\widehat{y}}_{i}\right)}^{2}$$

In Eq. 5, n is the total number of samples, ${y}_{i}$ is the true activity value of the i-th sample, and ${\widehat{y}}_{i}$ is the predicted activity value for the i-th sample. By minimizing the MSE, the predictor updates the model parameters to improve activity predictions, gradually reducing the loss. The predictor hyperparameters and training protocol for each dataset are reported in Supplementary Information Section 1 and Table S2.

### Supervised fine-tuning with experience replay and tournament selection

The SFT phase utilizes an iterative refinement framework that integrates the pre-trained generator and predictor. The core components are experience replay and multi-stage tournament selection. Experience memory serves as a repository for molecules. Initially, the experience memory is populated with unique, chemically valid molecules produced by the generative model before the fine-tuning loop begins. In the fine-tuning loop, the final winning molecules from the tournament selection are stored in the experience memory. The competitor pool is formed by merging the SMILES sampled from the generator with an equal number sampled from the experience memory. Tournament selection is used as a strategy to select superior molecules from the pool of sampled candidates [15]. At each stage, molecules compete based on specific criteria, and the winners survive to the next stage. The tournament selection process consisted of three stages: the first stage evaluated the predicted binding affinity of the molecules, the second stage assessed the negative log-likelihood from the generator, and the third stage calculated the positive log-likelihood from the prior model. In each stage, two molecules were randomly selected from the pool, and the molecule with the higher score was chosen as the winner. The winner advanced to the next stage, and the loser was excluded, so that only half of the molecules survive at each stage. This iterative process enables the generator to focus on molecules with high binding affinities. The framework allows the generator to explore new molecular structures while maintaining structural features that contribute to high biological activity.

Fine-tuning was conducted for a fixed budget of 30 epochs. This budget was determined based on the turnover dynamics of the experience replay mechanism. At each epoch, we sample 50,000 scaffold-conditioned SMILES; the competitor pool is then formed by combining the resulting unique valid molecules with a fixed-size sample drawn from the experience memory. A three-stage tournament selection retains one-eighth of the pool as winners, which are used to update the experience memory (capacity: 50,000) and to guide the supervised update of the generator. Under this mechanism, the memory contents are refreshed multiple times over the 30-epoch budget, allowing the generator to progressively focus on high-affinity regions while avoiding excessive computational cost.

During fine-tuning, scaffold-conditioned generation was performed using a fixed set of five reference scaffolds instead of conditioning on a single scaffold. The reference scaffolds were compiled to span chemically distinct frameworks including heteroaromatic systems, benzofused heterocycles, and flexible diaryl and amine frameworks. This design choice was adopted to reduce the risk that optimization dynamics become dominated by a single scaffold topology and to minimize sensitivity to a specific scaffold choice. All fine-tuning hyperparameters and tournament selection settings are summarized in Supplementary Information Section 1 and Table S3.

### Evaluation metrics

Performance evaluation in de novo molecular design uses metrics that differ from those employed in traditional machine-learning tasks such as regression and classification. In this study, we used nine metrics, grouped into two categories: (1) generative quality metrics and (2) distribution similarity metrics. Generative quality metrics included validity, uniqueness, novelty, internal diversity, sphere exclusion diversity (SEDiv) and predicted bioactivity (PredAct), while distribution similarity metrics encompassed pairwise similarity (PwSim), Fréchet Chemnet distance (FCD), and optimal transport distance (OTD). The generative quality metrics are defined as follows:

6 $$Validity=\frac{V}{20000}$$

7 $$Uniqueness= \frac{U}{V}$$

8 $$Novelty= \frac{T}{U}$$

9 $$Internal\, Diversity= \frac{1}{{\left|{V}_{1000}\right|}^{2}} {\sum}_{{m}_{1}\in {V}_{1000}, {m}_{2}\in {V}_{1000}}1-sim\left({m}_{1},{m}_{2}\right)$$

Validity assesses whether the generated molecules are chemically valid; RDKit was used in this process [31]. We generated 20,000 SMILES and calculated the proportion (${\boldsymbol{V}}$) of the SMILES representing chemically valid structures. Uniqueness measures the diversity of the generated SMILES and is expressed as the ratio (U) of unique molecules among the valid set (V). A low uniqueness score suggests that the model repeatedly generates the same molecules, indicating a limited capacity for learning the distribution. Novelty is defined as the proportion (T) of valid, unique molecules that do not exist in the training dataset. A low novelty score indicates that the model is overfitting. Internal diversity assesses structural diversity within the generated SMILES. A total of 1000 molecules were randomly selected among the valid molecules, and the similarity between these molecules was calculated using the Tanimoto similarity. In Eq. 9, $sim({m}_{1}, {m}_{2})$ represents the Tanimoto similarity between molecules ${m}_{1}$ and ${m}_{2}$, calculated using their Morgan fingerprint with a radius of 2 and 2048 bits. ${V}_{1000}$ represents the subset of 1000 randomly selected molecules from V. SEDiv was computed using sphere exclusion on ECFP4 fingerprints (Morgan radius 2) with a fixed Tanimoto distance threshold. SEDiv is defined as the fraction of representative leader molecules selected from a randomly sampled subset of valid and unique generated molecules, where higher values indicate greater structural variety [42]. While internal diversity captures pairwise dissimilarity within a fixed-size sample, SEDiv captures the coverage of distinct scaffold clusters, providing a complementary perspective on molecular diversity. PredAct is defined as the average predicted bioactivity of molecules generated by the model. The distribution similarity metrics are defined as follows:

10 $$PwSim=\frac{1}{\left|{V}_{1000}\right|\left|T\right|}{\sum}_{{m}_{1}\in {V}_{1000}, { m}_{2}\in T}sim\left({m}_{1},{m}_{2}\right)$$

11 $$FCD={|| {\mu}_{V} - {\mu}_{T} ||}^{2}+Tr({C}_{V} + {C}_{T} - {2({C}_{V}{C}_{T})}^{1/2})$$

12 $$OTD = argmi{n}_{T\in R}{\sum}_{{x}_{i}\in A , {y}_{j}\in B}{T}_{ij}dist\left({x}_{i},{y}_{j}\right)$$

13 $$dist\left({x}_{i},{y}_{j}\right)={10}^{1-sim\left({x}_{i},{y}_{j}\right)}-1$$

PwSim measures the average pairwise similarity between the generated SMILES and active molecules in the test dataset (Eq. 10). In this equation, T denotes the set of target active molecules in the test set. The FCD measures the difference between two probability distributions, specifically between the generated molecule set and the target molecule set, using the Fréchet distance (Eq. 11). This metric quantifies the dissimilarity between the two sets by comparing the means and covariances of their distributions, assuming both follow Gaussian distributions. Here, ${\mu}_{V}$ and ${\mu}_{T}$ represent the means of the feature vectors for the generated molecule set V and the target molecule set T, respectively, while ${C}_{V}$ and ${C}_{T}$ represent their covariance matrices. Tr represents the trace of a matrix, which is the sum of its diagonal elements. The OTD calculates the optimal transport cost between the two probability distributions of the generated molecule set and the target molecule set, thereby measuring the distance between the sets. This method is defined in terms of the similarity between probability distributions. Higher similarity results in lower OTD values (Eq. 12). In this formulation, ${T}_{ij}$ represents the amount of mass transported from molecule ${x}_{i}$ to molecule ${y}_{j}$. R is the set of all possible transport plans between the generated molecule set A and the target active set B. $dist ({x}_{i}, {y}_{j})$ represents the distance used for the OTD calculation. The performance evaluation metrics used in this study are similar to those used in previous studies [2, 15]. Notably, except for the OTD and FCD, higher metric values indicate improved performance.

## Results

### Performance of the pre-trained generator and predictor

We evaluated the performance of the pre-trained generative and predictive models before fine-tuning. Specifically, we enhanced the basic SMILES generation capability of the generative model by experimenting with different loss functions and applying a multi-scale attention mechanism. We also adjusted the temperature parameter to control sampling stochasticity and identified the optimal balance between validity and uniqueness during the SMILES generation. We conducted an ablation study to evaluate the impact of each module on the ability of the generator. The results provided insight into how these modifications affected the performance of the model in terms of validity, uniqueness, and novelty.

We randomly selected 50 scaffolds from the test set as input conditions to comprehensively evaluate the generalization capability of the model. This random selection strategy was adopted to avoid selection bias and to provide an unbiased assessment of the model's ability to generalize across diverse scaffold structures. No additional filtering criteria were applied, ensuring that the evaluation reflects the model's performance on scaffolds with varying structural complexity and chemical properties. We generated 10,000 SMILES for each scaffold and assessed the resulting molecules using the chosen metrics. To ensure statistical robustness, this generation and evaluation process was repeated five times with different random seeds, and the results are reported as mean ± standard deviation. The performance for the SMILES generated from each scaffold is presented in Table 2, showing the top 10 scaffolds. The two-dimensional structures of all scaffolds used in Tables 2 and 5 are provided in Supplementary Information Fig. S5. Across all scaffolds, the pre-trained generator achieved a validity value greater than 0.9, a uniqueness value greater than 0.9, and a novelty score of 1.0. Additionally, the pre-trained generator achieved an average validity of 0.968, uniqueness of 0.966, and novelty of 1.0. Notably, the performance of the generator varied significantly depending on the input scaffold. This variation occurred because each scaffold has distinct structural and chemical properties that affect the complexity and feasibility of generating valid molecules. Scaffolds with more available attachment sites and open valences provided the model with a larger space of valid decoration options, leading to higher validity and uniqueness values in the generated SMILES. Conversely, scaffolds with limited decoration sites due to ring closure or unsaturation constrained the feasible molecular space, thereby reducing generation performance. For instance, the scaffold O = C(Cc1ccccc1)NCc1ccccc1 contains two phenyl rings connected through a benzyl–amide linkage, providing multiple positions at ortho, meta, and para sites where substituents can be appended. This scaffold achieved a higher validity of 0.98 and uniqueness of 0.983. In contrast, the scaffold O = C1CCC(c2ccc(NCc3ccccc3)cc2) = NN1 features ring closure and unsaturation within the ring system, which saturates the atoms involved and limits the available positions for decoration. Consequently, the scaffold O = C1CCC(c2ccc(NCc3ccccc3)cc2) = NN1 achieved a lower validity value of 0.958 and a uniqueness score of 0.954. This result supports the assertion that the number of available attachment points and open valences in a scaffold positively affects the performance of the generator in terms of validity and uniqueness. We further evaluated the generator on 10 scaffolds that were completely absent from the training dataset to assess the model's generalization capability. The generator achieved comparable performance on these unseen scaffolds, with average validity of 0.958, uniqueness of 0.965, and novelty of 1.0, demonstrating robust generalization capability. Detailed results are provided in Supplementary Information Section 6 and Table S5.

**Table 2:** Performance evaluation of SMILES generated for each scaffold

Scaffold	Validity	Uniqueness	Novelty
O = C(Cc1ccccc1)NCc1ccccc1	0.980 ± 0.002	0.983 ± 0.002	1.000 ± 0.000
c1ccc(-c2ccnnc2)cc1	0.985 ± 0.001	0.944 ± 0.002	1.000 ± 0.000
c1ccc(C2 = NOCC2)cc1	0.968 ± 0.003	0.982 ± 0.001	1.000 ± 0.000
c1ccc(Cc2cc3c(CNC4CCCCC4)cccc3o2)cc1	0.964 ± 0.003	0.957 ± 0.002	1.000 ± 0.000
c1ccc(OCCn2ccnc2)c(CNCc2nccs2)c1	0.961 ± 0.004	0.975 ± 0.001	1.000 ± 0.000
C(= NCC(c1ccccc1)N1CCCCC1)c1ccccc1	0.967 ± 0.001	0.961 ± 0.003	1.000 ± 0.000
O = C(Nc1ccccc1)NC1CCC(OCc2ccccc2)CC1	0.981 ± 0.002	0.955 ± 0.003	1.000 ± 0.000
O = C(COC(= O)c1ccccc1)Nc1ccccc1N1CCOCC1	0.962 ± 0.001	0.962 ± 0.001	1.000 ± 0.000
O = C(Nc1ccccc1)c1cccc(N2C = CNN2)c1	0.954 ± 0.002	0.991 ± 0.001	1.000 ± 0.000
O = C1CCC(c2ccc(NCc3ccccc3)cc2) = NN1	0.958 ± 0.002	0.954 ± 0.004	1.000 ± 0.000
Average	0.968	0.966	1.0

We compared three generator variants, including a generator trained with cross-entropy as the loss function, a generator with multi-scale attention using scales 1 to 5, and a generator without multi-scale attention, to isolate the effects of the loss function and multi-scale attention. The cross-entropy variant achieved slightly higher validity but markedly lower uniqueness, whereas extending the multi-scale attention beyond the optimal range or removing it reduced both metrics. All models maintained a novelty score of 1.0. Details are provided in Supplementary Information Section 3 and Fig. S2. Additionally, we conducted experiments to determine the optimal temperature that achieves the best trade-off between validity and uniqueness. The temperature parameter modulates the probability distribution over candidate tokens during generation, thereby controlling randomness in sampling. As temperature increases, randomness rises and uniqueness improves at the expense of validity, whereas lower temperatures have the opposite effect. In our experiments, a temperature of 0.9 provided the most balanced result. The probability profiles and full results are provided in Supplementary Information Section 4, Fig. S3, and Table S4. Additionally, we analyzed the learning dynamics of scaffold awareness during pre-training. The scaffold retention rate increased progressively from 86 to 98% over 10 epochs, demonstrating that scaffold awareness strengthens as the model converges. Detailed analysis is provided in Supplementary Information Section 5 and Fig. S4.

**Fig. 4:**
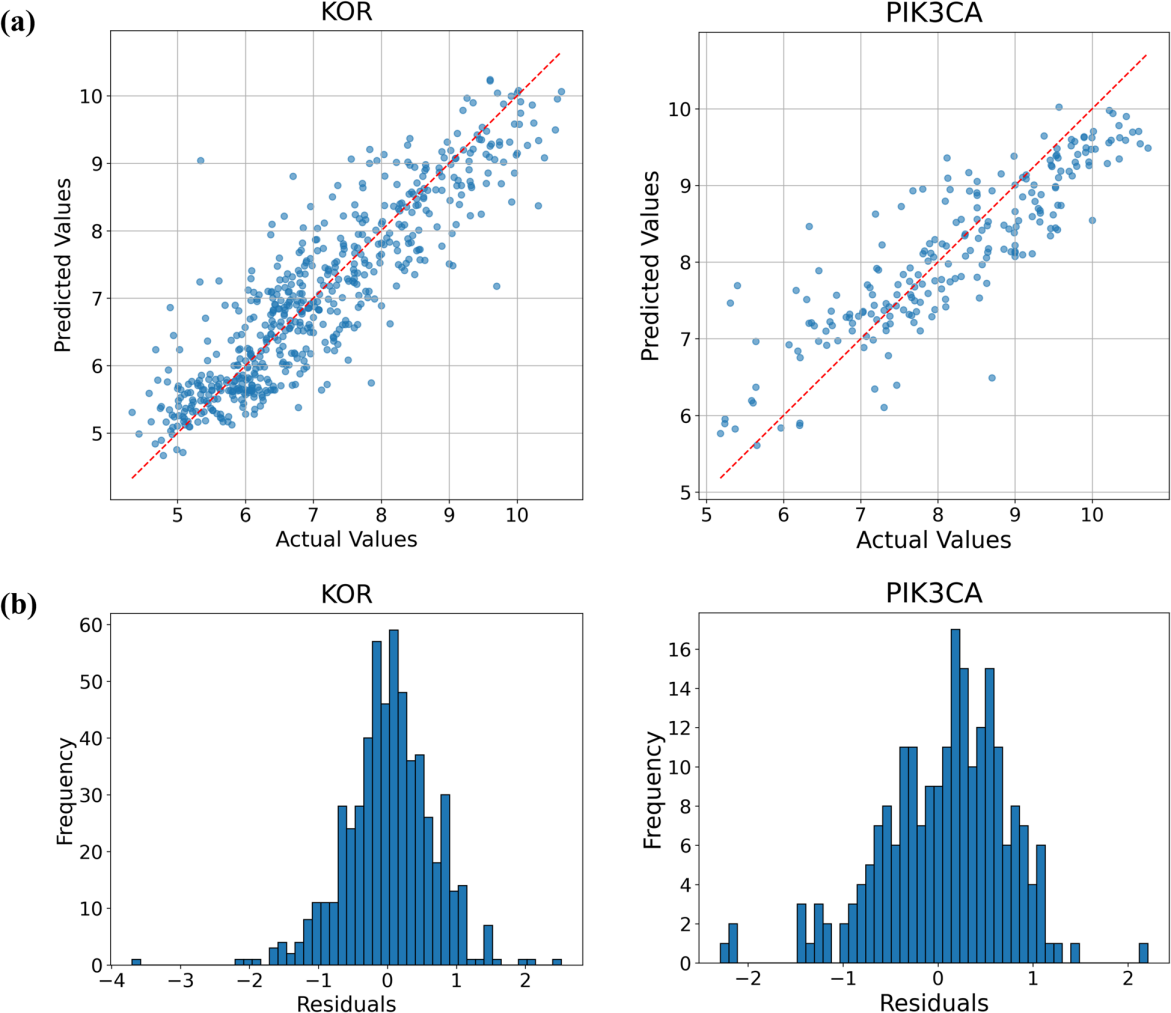
Visualization of the predictor performance on the KOR and PIK3CA datasets. **a**, **b** Predicted and actual biological activity values for the test datasets of the KOR and PIK3CA. The x-axis represents the actual values, and the y-axis represents the predicted values. The red line indicates the ideal case where the predicted values perfectly match the actual values. **c**, **d** Distribution of residuals (differences between actual and predicted biological activity values) for the KOR and PIK3CA datasets. The x-axis represents the residual values, and the y-axis represents the frequency of occurrence for each residual value

The pre-trained predictor for PIK3CA achieved an MSE of 0.444 and an R^2^ of 0.744, while the MSE for the KOR was 0.416 and the R^2^ was 0.788. Following this assessment of the predictive models for the PIK3CA and KOR, we performed a visual analysis to demonstrate their performances (Fig. 4). Figure 4a and b show scatter plots of the predicted versus actual values, demonstrating the correlation and predictive accuracy for each protein. In both datasets, the predicted and actual biological activity values show a high overall correlation. The data points are densely clustered around the red solid line, indicating that the models accurately predict the actual values. Figure 4c and d show residual histograms, illustrating the distribution of the prediction errors and the consistency of the predictors. In both datasets, the residuals are symmetrically distributed around a mean of zero, indicating that the predictions by the models are generally unbiased. For the KOR, the distribution is narrower, with over 95% of the residuals distributed between − 1 and 1; for PIK3CA, over 95% of the residuals are distributed between − 1.5 and 1.5.

We performed fivefold cross-validation to verify the robustness of the predictor across different data splits. The GAT predictor achieved mean test R^2^ values of 0.788 ± 0.028 for KOR and 0.744 ± 0.027 for PIK3CA, with low standard deviations indicating consistent performance regardless of the specific train/test partition. Detailed cross-validation results are provided in Supplementary Information Section 6 and Table S6.

### Performance of the fine-tuned generative model

We evaluated our Scaffold-Aware Transformer against three baseline models: LOGICS, PromptSMILES, and LibINVENT [15, 22, 43]. LOGICS shares the same pre-training and fine-tuning framework as our model but lacks multi-scale attention. PromptSMILES uses scaffold SMILES as prefix prompts for a de novo RNN generator optimized through reinforcement learning. LibINVENT operates through its LibINVENT module as a hard-constraint scaffold decorator that restricts generation to predefined attachment points. All baselines were trained using identical scoring components consisting of our GAT-based bioactivity predictor and QED drug-likeness. Five independent experiments with different random seeds were performed, each generating 20,000 SMILES, and results are reported as mean ± standard deviation. The results are summarized in Tables 3 and 4.

**Table 3:** Performance evaluation of fine-tuned generative models on the KOR dataset

Performance metrics	Scaffold-aware transformer	LOGICS	PromptSMILES	LibINVENT
Validity	0.9802 ± 0.000	0.9664 ± 0.002	0.9801 ± 0.001	0.9785 ± 0.001
Uniqueness	0.9865 ± 0.000	0.9994 ± 0.000	0.9976 ± 0.002	0.9823 ± 0.000
Novelty	0.9998 ± 0.000	0.9774 ± 0.000	0.9801 ± 0.000	0.9999 ± 0.000
Internal diversity	0.8477 ± 0.000	0.8764 ± 0.001	0.8626 ± 0.001	0.7945 ± 0.001
SEDiv	0.4308 ± 0.001	0.7771 ± 0.004	0.7180 ± 0.001	0.2490 ± 0.003
PredAct	6.5605 ± 0.002	6.4556 ± 0.006	6.4397 ± 0.002	6.3713 ± 0.003
PwSim	0.1326 ± 0.000	0.1260 ± 0.000	0.1246 ± 0.000	0.1322 ± 0.000
FCD	25.5467 ± 0.022	21.2631 ± 0.072	26.6456 ± 0.022	23.4803 ± 0.042
OTD	5.4578 ± 0.003	5.0830 ± 0.015	5.2516 ± 0.004	5.4780 ± 0.007

**Table 4:** Performance evaluation of fine-tuned generative models on the PIK3CA dataset

Performance metrics	Scaffold-aware transformer	LOGICS	PromptSMILES	LibINVENT
Validity	0.9771 ± 0.021	0.9704 ± 0.001	0.9781 ± 0.009	0.9881 ± 0.001
Uniqueness	0.9917 ± 0.001	0.9989 ± 0.000	0.9990 ± 0.001	0.9904 ± 0.000
Novelty	0.9998 ± 0.002	0.9744 ± 0.001	0.9725 ± 0.001	0.9997 ± 0.000
Internal diversity	0.8441 ± 0.000	0.8689 ± 0.001	0.8780 ± 0.000	0.7920 ± 0.001
SEDiv	0.4564 ± 0.004	0.8631 ± 0.001	0.8420 ± 0.001	0.2540 ± 0.001
PredAct	8.0574 ± 0.002	7.5792 ± 0.005	7.3561 ± 0.002	7.7592 ± 0.002
PwSim	0.1092 ± 0.000	0.1124 ± 0.000	0.1139 ± 0.000	0.1157 ± 0.000
FCD	40.8367 ± 0.021	38.7771 ± 0.192	32.8511 ± 0.027	32.7856 ± 0.031
OTD	5.9646 ± 0.001	5.7650 ± 0.021	5.6080 ± 0.011	5.9712 ± 0.002

Our model achieved the highest PredAct on both targets, reaching 6.5605 for KOR and 8.0574 for PIK3CA, exceeding all baselines. Validity remained competitive at 0.9802 and 0.9771, and novelty was notably high at 0.9998 for both targets. LibINVENT showed the lowest SEDiv of 0.249 and 0.254, and the lowest internal diversity of 0.7945 and 0.7920 for KOR and PIK3CA respectively. PromptSMILES maintained balanced diversity metrics but achieved lower PredAct, particularly for PIK3CA at 7.3561. LOGICS achieved the lowest FCD and OTD values across both targets, along with the highest uniqueness and SEDiv scores.

We tracked multiple performance metrics across 30 fine-tuning iterations to verify the effectiveness of the fine-tuning procedure. The predicted activity score consistently improved throughout training, confirming that the tournament-based selection mechanism effectively guides the generator toward higher-affinity molecules. Meanwhile, validity, uniqueness, and novelty remained stable with minor fluctuations, indicating that molecular quality is preserved during optimization. The detailed optimization curves are provided in Supplementary Information Section 7 and Fig. S6.

We assessed the drug-likeness and synthetic accessibility of the 15,000 molecules generated by the fine-tuned model using QED and SAS [44, 45]. For the KOR, around 20% of all molecules had a QED score above 0.6, and about 99% had an SAS below 5. For PIK3CA, around 46% had a QED score above 0.6, and about 99% had an SAS below 5. Detailed summaries and representative molecules from different QED bins are provided in Supplementary Information Section 8, Fig. S7 and Fig. S8.

We conducted a chemical space analysis using t-SNE dimensionality reduction to characterize the applicable chemical space of the proposed framework. The pre-trained generator produces molecules that substantially overlap with the training data distribution, while the fine-tuned models for KOR and PIK3CA extend into target-specific regions while maintaining coverage of the training chemical space. This result demonstrates that the fine-tuning process effectively guides molecular generation toward biologically relevant chemical space while preserving the capacity for structural novelty. The detailed chemical space analysis is provided in Supplementary Information Section 9 and Fig. S9. Additionally, we performed distance analysis to quantify how far the generated molecules are from the training data. The mean nearest-neighbor Tanimoto similarity ranged from 0.297 to 0.316 across models, and FCD values confirmed that fine-tuned models explore more distant regions of chemical space compared to the pre-trained generator. These results are presented in Supplementary Information Section 9 and Table S7.

We performed external validation using three independent prediction models to verify that the predicted activities are not artifacts of our GAT-based predictor. Random Forest Regressor, feed-forward neural network, and graph convolutional network were trained on the same data splits and applied to the fine-tuned generated molecules. All independent predictors consistently estimated activities within the high-affinity range, with mean predictions aligning closely with the GAT results reported in Tables 3 and 4. Detailed results are provided in Supplementary Information Section 10, Fig. S10, and Table S8.

### Scaffold fidelity under scaffold-conditioned generation

We quantified scaffold fidelity to verify whether the generator is genuinely scaffold-aware. For each reference scaffold used in Table 2, we generated 10,000 valid molecules under identical sampling conditions, and fidelity was evaluated using three complementary criteria: (i) exact scaffold substructure retention, defined as the fraction of valid generated molecules containing the input scaffold as an RDKit substructure; (ii) Murcko similarity rate, defined as the fraction whose Bemis–Murcko scaffold exhibits a Morgan fingerprint Tanimoto similarity of at least 0.6 to the input Murcko scaffold; and (iii) Murcko analog rate, defined as the fraction that is not an exact Murcko match but whose Bemis–Murcko scaffold exhibits a Morgan fingerprint Tanimoto similarity of at least 0.6 to the input Murcko scaffold [31]. We selected τ = 0.6 as the similarity threshold based on prior work that adopted this value as the boundary for high structural similarity [46, 47]. The analog criterion captures soft-constraint compliance, where the model preserves a closely related scaffold family even when the exact input scaffold is not reproduced.

Scaffold fidelity varied across scaffolds: some scaffolds achieved high exact substructure retention exceeding 0.85, whereas others showed reduced exact retention below 0.05. In cases where exact retention was lower, the Murcko similarity rate and mean Murcko similarity remained substantial, indicating that the generator preserves core scaffold similarity under soft conditioning even when hard constraints are not strictly enforced. The scaffold fidelity results are presented in Table 5. The two-dimensional depictions of these scaffolds are provided in Supplementary Information Fig. S5.

**Table 5:** Scaffold fidelity evaluation of generated SMILES

Scaffold	Exact	Similarity (τ = 0.6)	Analog (τ = 0.6)	Similarity mean
O = C(Cc1ccccc1)NCc1ccccc1	0.865	0.883	0.019	0.929
c1ccc(-c2ccnnc2)cc1	0.466	0.466	0.000	0.657
c1ccc(C2 = NOCC2)cc1	0.694	0.695	0.001	0.805
c1ccc(Cc2cc3c(CNC4CCCCC4)cccc3o2)cc1	0.044	0.201	0.157	0.436
c1ccc(OCCn2ccnc2)c(CNCc2nccs2)c1	0.0003	0.012	0.009	0.265
C(= NCC(c1ccccc1)N1CCCCC1)c1ccccc1	0.543	0.562	0.019	0.683
O = C(Nc1ccccc1)NC1CCC(OCc2ccccc2)CC1	0.409	0.493	0.084	0.692
O = C(COC(= O)c1ccccc1)Nc1ccccc1N1CCOCC1	0.270	0.718	0.448	0.681
O = C(Nc1ccccc1)c1cccc(N2C = CNN2)c1	0.031	0.039	0.008	0.402
O = C1CCC(c2ccc(NCc3ccccc3)cc2) = NN1	0.527	0.636	0.111	0.729

We further analyzed scaffold retention for PromptSMILES and LibINVENT using the same 10 scaffolds and metrics. PromptSMILES achieved exact Murcko retention of 1.0 for all scaffolds but showed low Murcko similarity for simple scaffolds such as c1ccc(-c2ccnnc2)cc1, where the similarity dropped to 0.031, indicating substantial structural modifications beyond the scaffold core. LibINVENT achieved exact retention of 1.0 for 9 of 10 scaffolds, but one scaffold failed entirely with 0.0 exact retention due to incompatible attachment point decomposition. The detailed per-scaffold retention results for both baselines are provided in Supplementary Information Section 11, Table S9 and Table S10.

### Attention-based substructure analysis

**Fig. 5:**
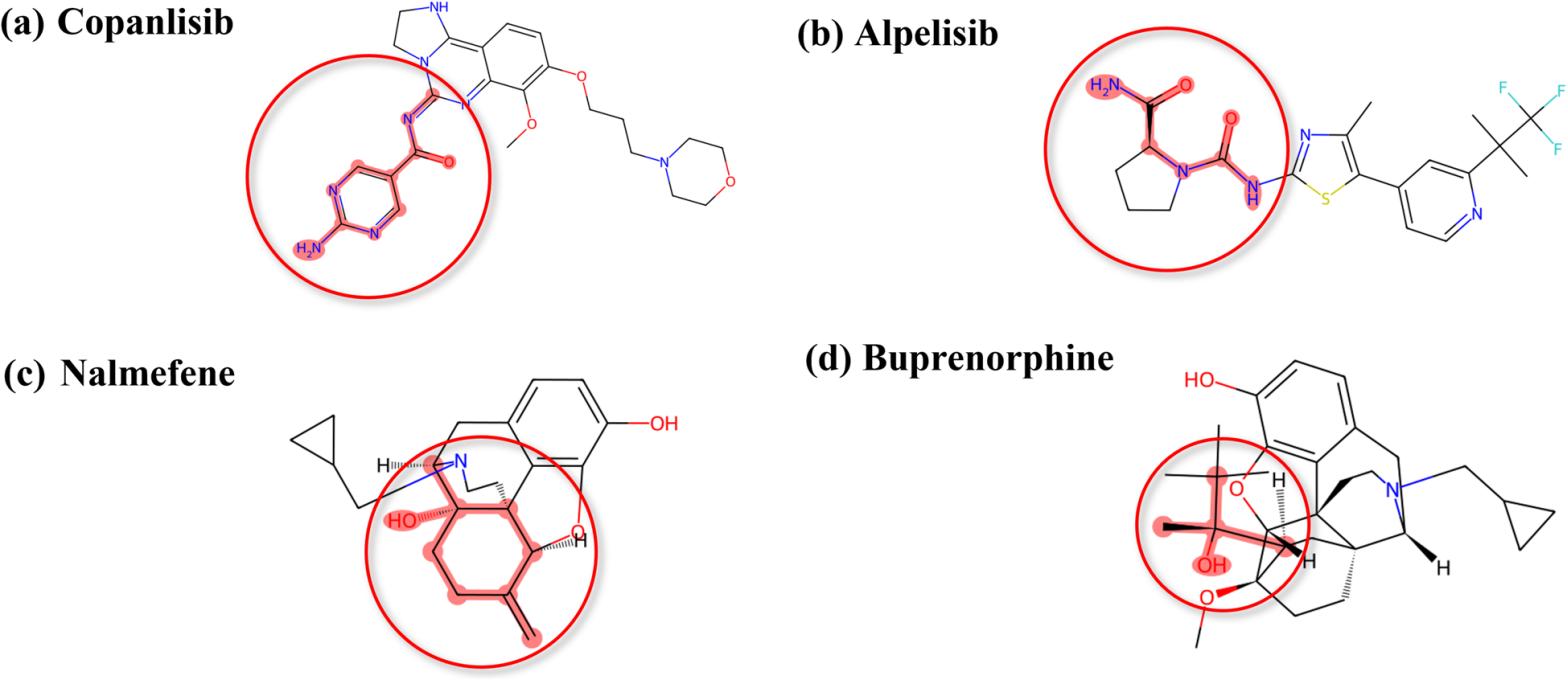
Attention-based substructure analysis of the reference drugs for PI3Kα and the KOR. **a** Copanlisib, **b** Alpelisib, **c** Nalmefene, and **d** Buprenorphine. Red-highlighted regions indicate substructures with high attention scores from the graph attention network (GAT)

We identified substructures associated with the prediction of the model using attention coefficients from a GAT. This approach offers substructure-level interpretability for the target and identifies molecular motifs that the model considers most important for predicting binding affinity. We cross-validated the highlighted substructures with prior studies on mechanisms of action, including reported binding modes and pharmacophores, to support the interpretation of the model. We performed this analysis on the drugs that target PIK3CA, Copanlisib and Alpelisib, as well as the drugs that target the KOR, Nalmefene and Buprenorphine. The attention-highlighted substructures for all drugs are presented in Fig. 5. All medications used in the analysis have received approval from the United States Food and Drug Administration (FDA) [48–50].

The attention analysis for Copanlisib highlighted the aminopyrimidine group linked to the dihydroimidazoquinazoline core. In the crystallographic binding model, this group occupies the affinity pocket and forms a three-point hydrogen bond network in which the exocyclic amine donates to Asp836 and Asp841, and a ring nitrogen accepts a hydrogen bond from Lys833 [51]. The fused tricyclic core also engages the hinge valine through a ring nitrogen and anchors the ligand within the ATP pocket. As reported in previous studies, the morpholinopropyl side chain extends toward the solvent, primarily improving solubility with a limited direct contribution to binding. Findings from the lead optimization indicate that the aminopyrimidine is the preferred substitution for potency, the methoxy preserves the pocket fit, and the morpholinylpropoxy group was optimized to tune properties and pharmacokinetics [52].

For Alpelisib, the attention analysis highlighted the proline-derived carboxamide linked through a urea and the adjacent 2-aminothiazole motif. Prior structure–activity relationship studies have shown that Alpelisib donates and accepts hydrogen bonds to Gln859 and to the backbone carbonyl of Ser854, engages the hinge Val851, and makes a water-mediated contact from Asp810 and Asp933 to the pyridine nitrogen, with the charged Lys802 positioned near the trifluoromethyl group [48, 53]. Complementary SAR and docking studies have further indicated that contacts with Gln859, Ser854, and Val851 are central to selectivity and binding, and that the 2-aminothiazole scaffold with an L-proline-derived carboxamide promotes selectivity for the PI3Kα subtype [48, 54, 55].

The three-dimensional binding modes of Copanlisib and Alpelisib with labeled residues are provided in Supplementary Information Section 12 and Fig. S11, confirming that the attention-highlighted regions correspond to direct protein–ligand contacts observed in crystallographic structures. The attention-highlighted regions differ between Copanlisib and Alpelisib because these inhibitors employ distinct binding modes. Notably, the hinge-binding domain received lower attention weights in both cases despite its importance for kinase inhibition. We attribute this to the conserved nature of hinge-binding interactions, which limits their discriminative power for distinguishing affinity differences between ligands. The GAT model instead prioritizes selectivity-determining regions that vary between inhibitors and better explain the observed affinity differences in the training data.

The attention analysis for Nalmefene highlights the phenolic ring and the adjacent hydroxyl-substituted ring. According to prior docking studies on the KOR, Nalmefene forms three hydrogen bonds in the KOR site. The hydroxyl group of Tyr139 forms a hydrogen bond with a ligand oxygen, and the ligand hydroxyl forms hydrogen bonds with the nitrogen of Gln115 and the oxygen of Asp138 [50]. In our visualization, the highlighted substructure captures the hydroxyl-rich region that can interact with Gln115 and Asp138, whereas the attention of the model does not prioritize the Tyr139 contact. Therefore, the model regards this highlighted moiety as the key motif that most strongly explains the binding affinity of Nalmefene to the KOR.

The Buprenorphine attention analysis highlighted the tertiary-alcohol motif and focused on the oxygen-containing segment. Prior docking work on the KOR has reported a single hydrogen bond in which the hydroxyl hydrogen of the drug interacts with the oxygen of the Ile304 residue [50]. The highlighted substructure captures the alcohol functionality capable of mediating this contact.

## Discussion

This study contributes to the field of molecular generation by integrating scaffold-conditioned generation with an attention-based predictor [15, 26, 27]. Our approach demonstrated high validity and novelty in generating molecules, providing interpretable structure–activity explanations. These findings suggest that our model can generate new molecules with desired properties and has the potential to advance the design of de novo molecules.

Our ablation study shows that architectural choices and sampling control are key determinants of generation quality. Meanwhile, using focal loss with multi-scale scaffold attention improved uniqueness relative to cross-entropy, and a sampling temperature of 0.9 provided the most balanced trade-off between validity and uniqueness (Supplementary Information, Sections 3 and 4). Attention-based substructure analysis provided target-level interpretability. For PI3Kα, the highlighted motifs aligned with literature-reported contacts at Val851, Ser854, and Gln859. For the KOR, the model emphasized the hydroxyl-rich regions consistent with contacts to Gln115 and Asp138 for Nalmefene, and the Ile304 hydrogen bond for Buprenorphine, while deprioritizing the Tyr139 interaction. These comparisons with prior reports indicate that the features highlighted by the model are consistent with the previously reported chemical interactions, rather than incidental. The generated molecules also exhibited practical chemical properties. Most had SAS values within ranges consistent with feasible synthesis, and many exhibited moderate to high QED (Supplementary Information, Section 8).

The central contribution of our framework lies in integrating soft scaffold conditioning with supervised iterative optimization, which differs fundamentally from existing approaches. Hard-constraint methods such as LibINVENT guarantee scaffold preservation but limit chemical space exploration, and reinforcement learning optimization further exacerbates this by converging toward narrow high-scoring regions, as evidenced by the lowest diversity metrics in our comparative evaluation [22]. Prompt-based methods such as PromptSMILES lack explicit scaffold-aware training, resulting in inconsistent retention and weaker optimization [43]. Our multi-scale attention mechanism internalizes scaffold awareness as a learned feature during pre-training, while the tournament-based supervised fine-tuning optimizes bioactivity through direct selection, avoiding the reward hacking inherent in reinforcement learning. This combination achieved the highest predicted bioactivity on both targets while maintaining molecular diversity, a capability that neither hard-constraint decoration nor RL-based optimization alone provided.

Several limitations exist in this study. First, the performance of the model depends on the structural characteristics of the input scaffold. As demonstrated in Table 2, scaffolds with limited decoration sites due to ring closure or unsaturation showed lower validity and uniqueness compared to scaffolds with more available attachment points. This occurs because the model learns to append substituents at positions with open valences, and when such positions are scarce, the feasible molecular space becomes constrained. The observed scaffold-dependent variability in fidelity metrics is also consistent with these structural constraints. Exact retention tends to be higher for scaffolds that offer chemically feasible and diverse decoration patterns, whereas exact retention decreases for scaffolds with constrained topologies, including fused or highly saturated ring systems and limited substitution sites, where valid decorations are more restricted. Under such conditions, the model generates molecules that deviate from the exact query scaffold yet remain within a closely related scaffold family, as captured by the Murcko analog metrics. Therefore, reporting both exact retention and analog similarity provides a more comprehensive characterization of scaffold awareness under soft constraints. Second, there is a risk of overfitting to scaffold structures. Scaffold-based generation offers the advantage of maintaining desired core structures; however, the model may overfit to specific scaffold configurations, diminishing the diversity of generated molecules. Strategies such as using multiple scaffolds simultaneously or increasing scaffold diversity during training could mitigate this issue. Third, the generalization capability of the framework is limited by the evaluation on only two target datasets. Our framework was validated on KOR, a G-protein coupled receptor, and PIK3CA, a lipid kinase, which differ in protein family, binding site topology, and ligand chemotype. This provides an initial assessment of cross-target applicability, but two targets alone do not constitute conclusive evidence for generalization across the full diversity of druggable target classes, including ion channels, nuclear receptors, and proteases. The pre-trained generator is target-agnostic and trained on the GuacaMol dataset covering broad chemical space, while target specificity is introduced only during the SFT phase through the GAT-based predictor [28]. Extending the evaluation to additional targets such as DRD2 and EGFR, which represent further diversity in receptor family and mechanism of action, remains an important direction for future work. Fourth, our model showed lower pass rates on LillyMedChem-style chemical quality filters compared to baselines, with rates of 0.3686 for KOR and 0.2923 for PIK3CA (Supplementary Information Section 14, Table S12). This reflects a known trade-off between aggressive bioactivity optimization and chemical quality, as models achieving higher predicted activity tend to generate molecules with more reactive or flagged substructures [32, 56]. Incorporating chemical quality filters as additional scoring components during fine-tuning represents a straightforward extension enabled by the modular tournament selection framework.

The comparative evaluation against PromptSMILES and LibINVENT revealed informative trade-offs among scaffold-conditioning strategies [22, 43]. LibINVENT achieved the lowest diversity metrics across all methods, which reflects the inherent limitation of hard-constraint decoration where chemical exploration is confined to attachment-point substitutions. The reinforcement learning optimization further exacerbated this tendency by converging toward a narrow set of high-scoring R-group combinations. PromptSMILES maintained higher diversity but achieved the lowest predicted bioactivity for PIK3CA, suggesting that prompt-based conditioning provides weaker optimization pressure compared to explicit scaffold-aware training. LOGICS demonstrated favorable distributional similarity metrics, likely because its unconstrained generation remains closer to the prior distribution learned from theChEMBL-derived taining data [15, 29]. The trade-off between bioactivity optimization and distributional similarity observed in our model reflects a deliberate design choice: our model prioritizes generating potent scaffold-conditioned candidates through multi-scale attention and experience replay, rather than reproducing known chemical distributions. Hard-constraint methods such as LibINVENT are most suitable for late-stage lead optimization requiring exact scaffold preservation, while soft-constraint methods including our model are preferable for early-stage exploration where structural flexibility enables broader chemical space coverage combined with strong bioactivity optimization.

We also investigated whether SAR knowledge can transfer across different scaffolds and targets. Using held-out scaffolds from the MOSES dataset that were not present during fine-tuning, we compared molecules generated by the baseline, KOR fine-tuned, and PIK3CA fine-tuned generators, all scored by the same PIK3CA predictor [57]. The KOR fine-tuned generator consistently produced molecules with higher predicted activities than the baseline across all tested scaffolds, suggesting that fine-tuning captures generalizable SAR patterns that can partially transfer to unseen scaffolds and different targets. However, this analysis relies on in silico predictions rather than experimental validation, and the degree of transfer may vary depending on scaffold and target similarity. Detailed results are provided in Supplementary Information Section 13 and Table S11.

This study also suggests several ways for extension. First, the layered tournament selection is inherently modular, and additional drug discovery objectives such as ADMET properties can be incorporated by appending new tournament stages with corresponding predictive models or by replacing the single activity score with a composite scoring function that aggregates multiple endpoints. Either approach requires no modifications to the generator architecture or the experience replay mechanism, enabling the framework to simultaneously optimize potency and pharmacokinetic properties from the early stages of molecular design. Second, incorporating structure-based signals such as receptor-specific constraints and physics-guided priors into the training loop may further strengthen the link between attention-derived motifs and true binding determinants. Overall, our integration of scaffold-conditioned generation with an SFT framework represents a novel contribution to the field, potentially advancing the development of more effective drug discovery methods.

## Conclusion

This study introduced a scaffold-aware generative framework that integrates a transformer-based generator and a GAT-based predictor [26, 27]. Thus, by incorporating multi-scale attention mechanisms, our approach enables explicit scaffold control while exploring chemical diversity around user-specified core structures. An SFT framework with tournament selection and experience memory facilitates continuous optimization toward high-affinity, scaffold-consistent candidates [15]. The experimental results on the KOR and PIK3CA targets demonstrated that the proposed method achieves high validity and novelty while generating molecules with higher predicted biological activity compared to baseline approaches. Moreover, attention-based analysis of FDA-approved drugs revealed that the model highlights substructures consistent with known binding interactions, providing interpretable insights into structure–activity relationships. Meanwhile, the assessment of drug-likeness and synthetic accessibility revealed that the generated molecules exhibit practical chemistry profiles, with the majority showing favorable synthetic feasibility. Ablation studies confirmed that the combination of focal loss and multi-scale attention mechanisms significantly improves generation quality, and demonstrated that appropriate temperature control achieves an optimal balance between validity and uniqueness. This study presents a balanced and effective approach for generating novel bioactive molecules, highlighting the potential applicability of the proposed framework in drug discovery and material design. Future research is expected to contribute to the generation of more complex molecular structures and the expansion of models to consider a broader range of biological properties.

## Supplementary Information

Additional file 1

Additional file 2

Additional file 3

Additional file 4

Additional file 5

Additional file 6

Additional file 7

Additional file 8

Additional file 9

Additional file 10

Additional file 11

Additional file 12

## Data Availability

The code for the proposed model and data are available at https://github.com/bmil-jnu/Scaffold-Aware-Transformer
