# Supplementary figures and images for: Novel molecular design via a scaffold-aware transformer with multi-scale attention mechanisms
Source: J Cheminform. 2026 May 19;18:105. doi: 10.1186/s13321-026-01221-6 (PMC13425956; doi:10.1186/s13321-026-01221-6)

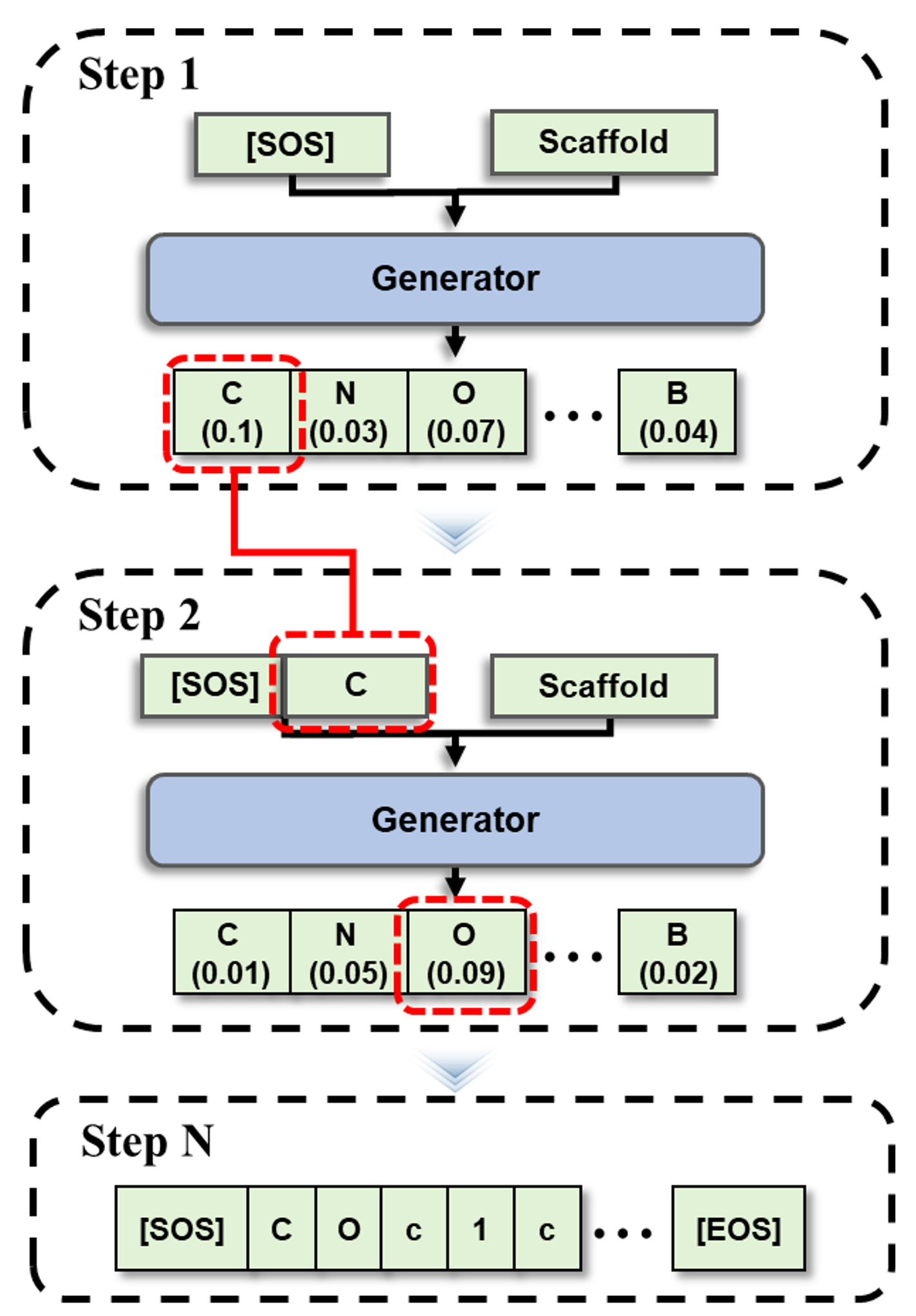

Supplement: Supplementary file 1 — Additional file 1 [file 13321_2026_1221_MOESM1_ESM.png]

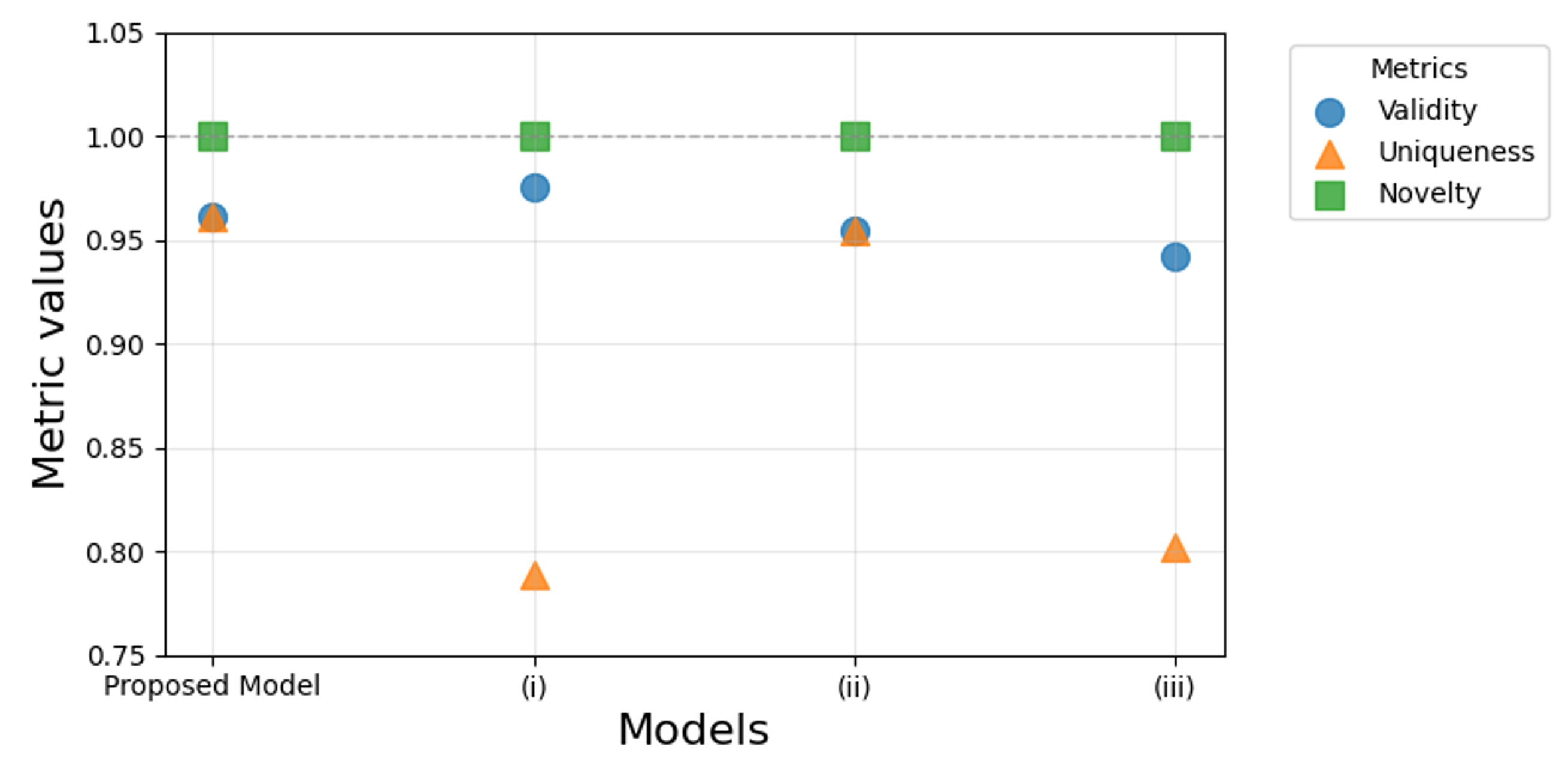

Supplement: Supplementary file 2 — Additional file 2 [file 13321_2026_1221_MOESM2_ESM.png]

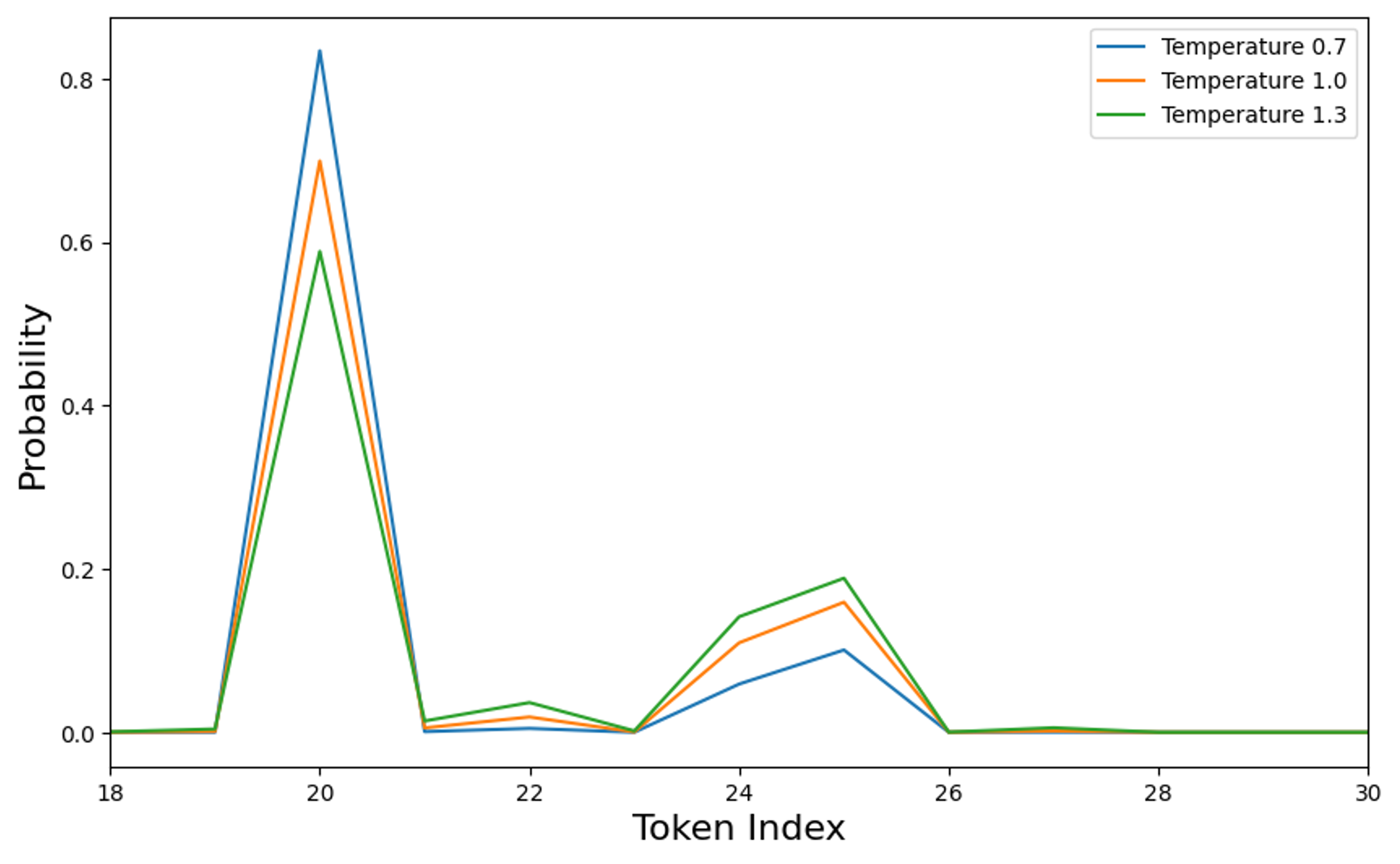

Supplement: Supplementary file 3 — Additional file 3 [file 13321_2026_1221_MOESM3_ESM.png]

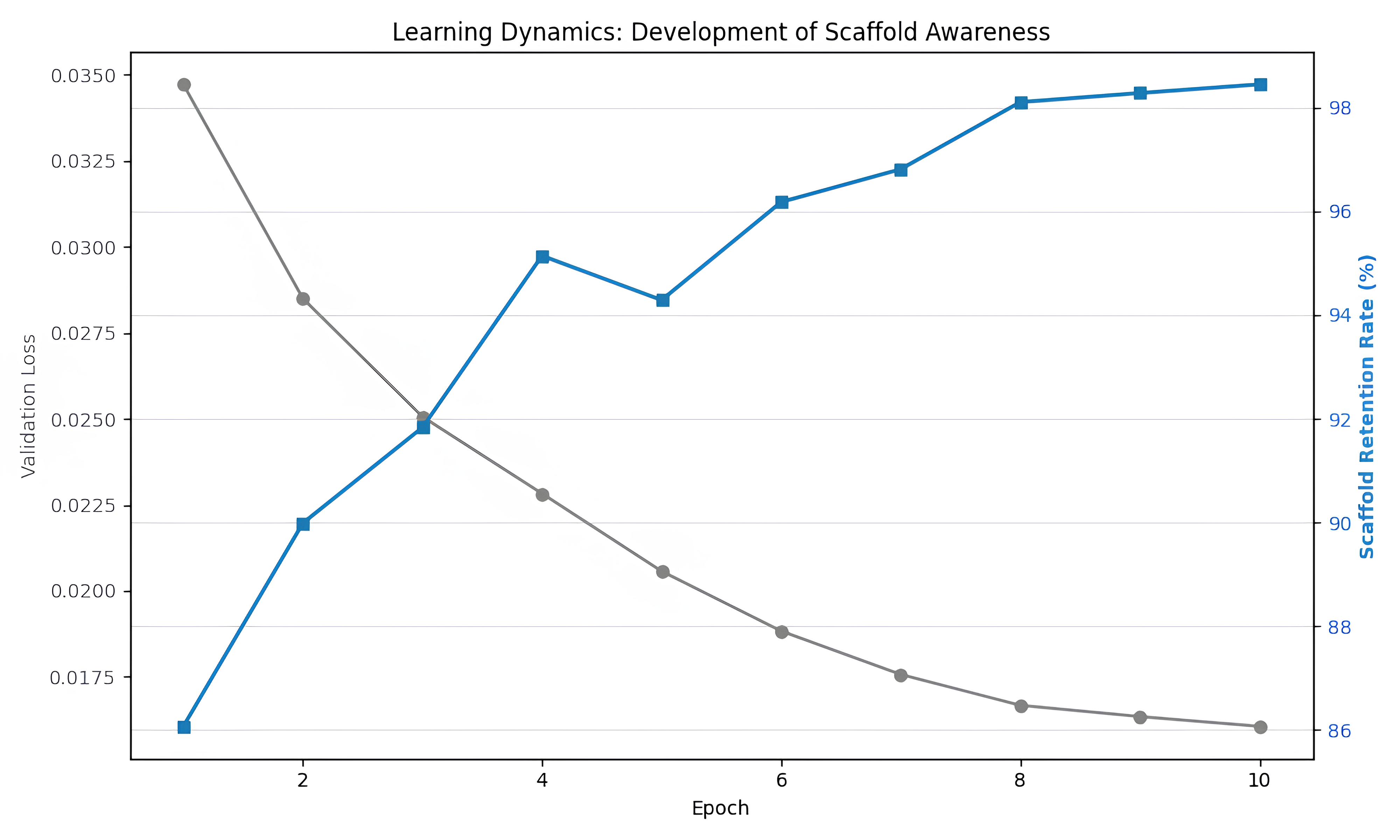

Supplement: Supplementary file 4 — Additional file 4 [file 13321_2026_1221_MOESM4_ESM.png]

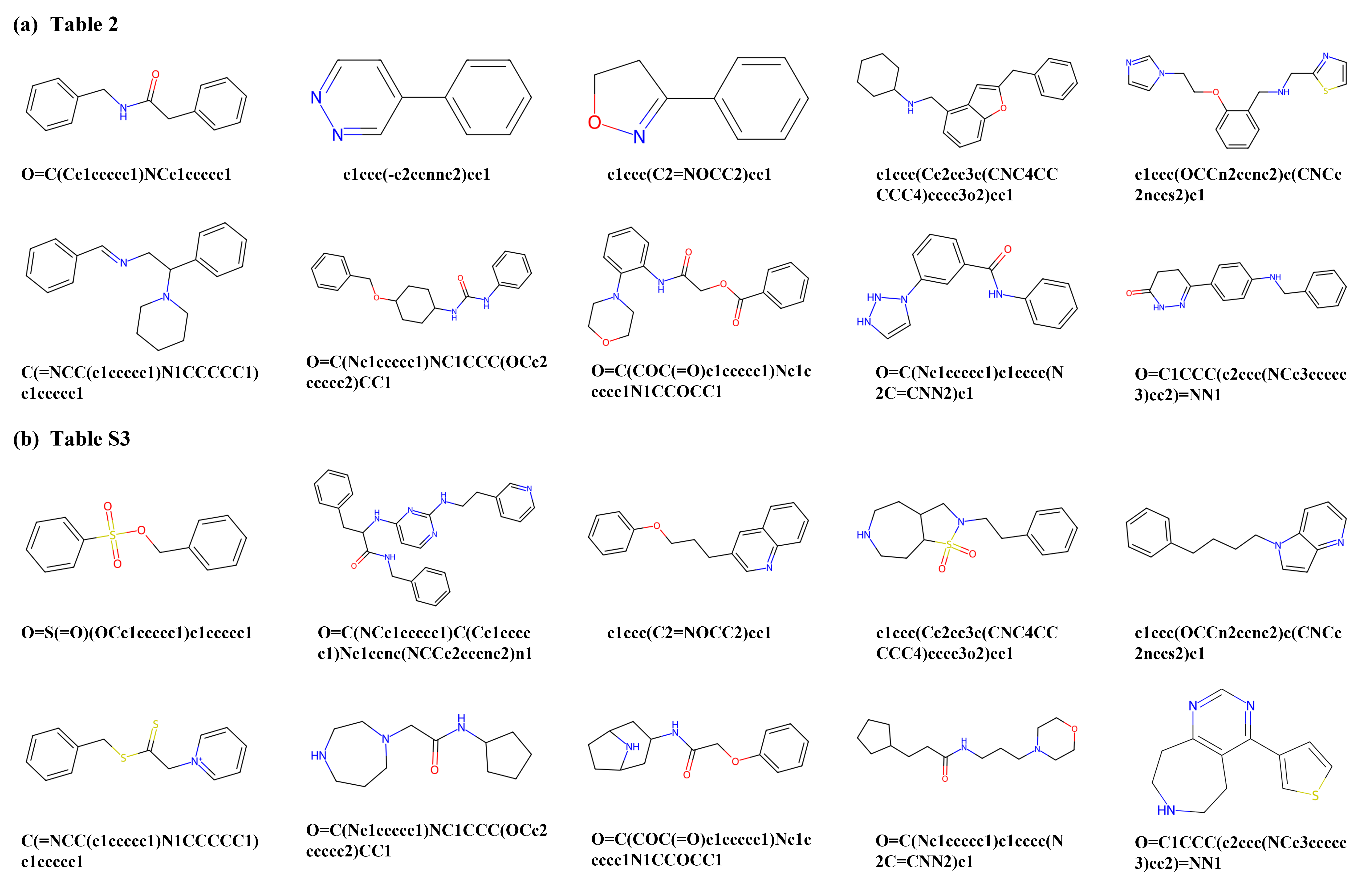

Supplement: Supplementary file 5 — Additional file 5 [file 13321_2026_1221_MOESM5_ESM.png]

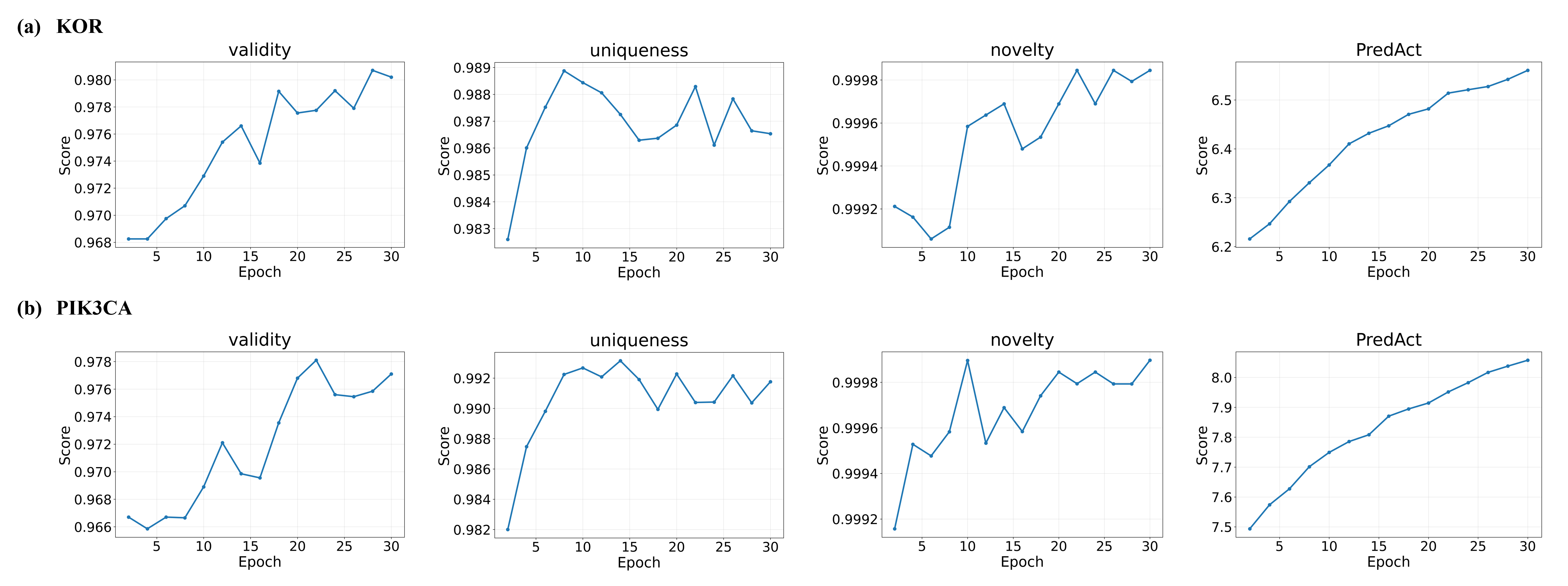

Supplement: Supplementary file 6 — Additional file 6 [file 13321_2026_1221_MOESM6_ESM.png]

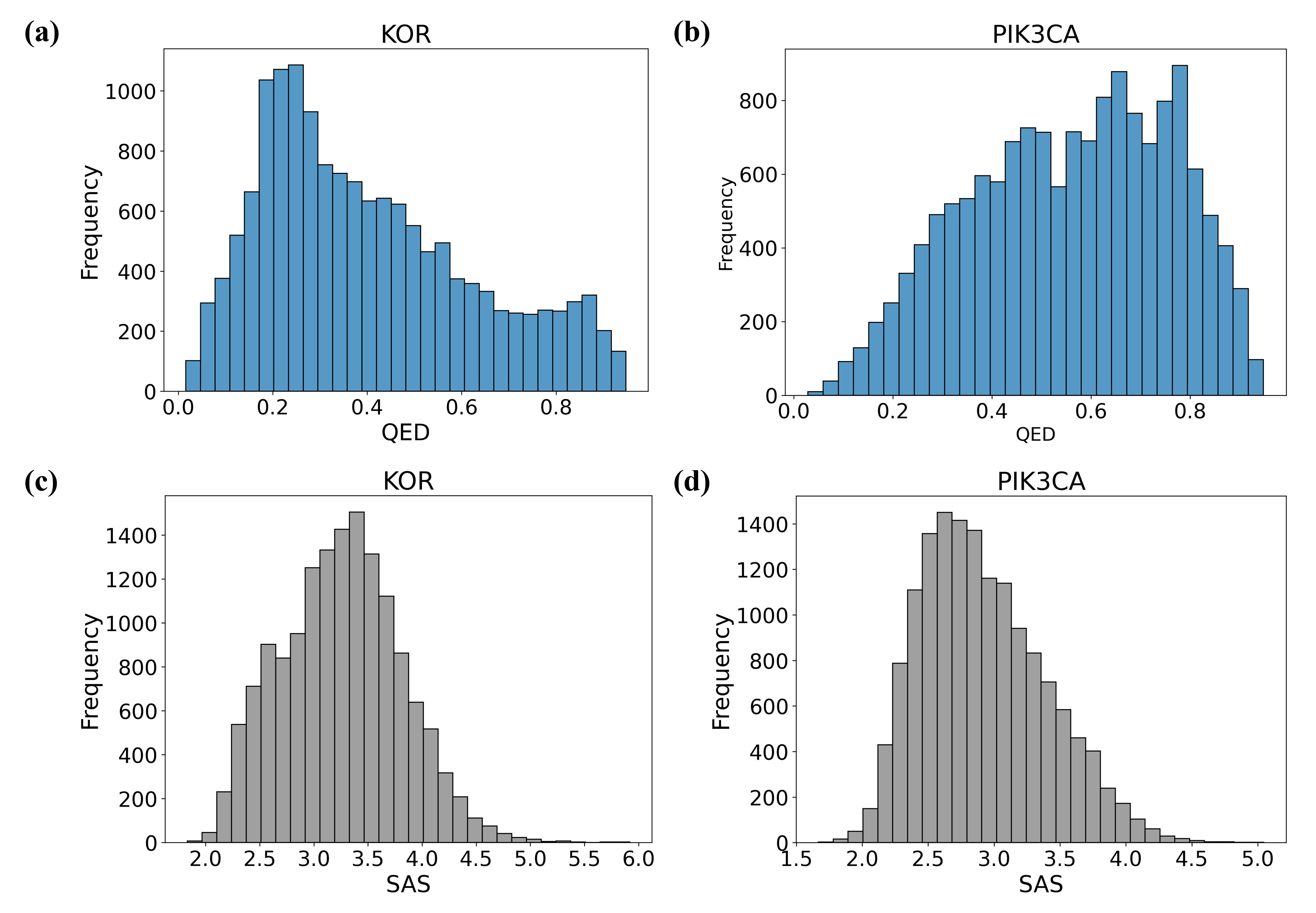

Supplement: Supplementary file 7 — Additional file 7 [file 13321_2026_1221_MOESM7_ESM.png]

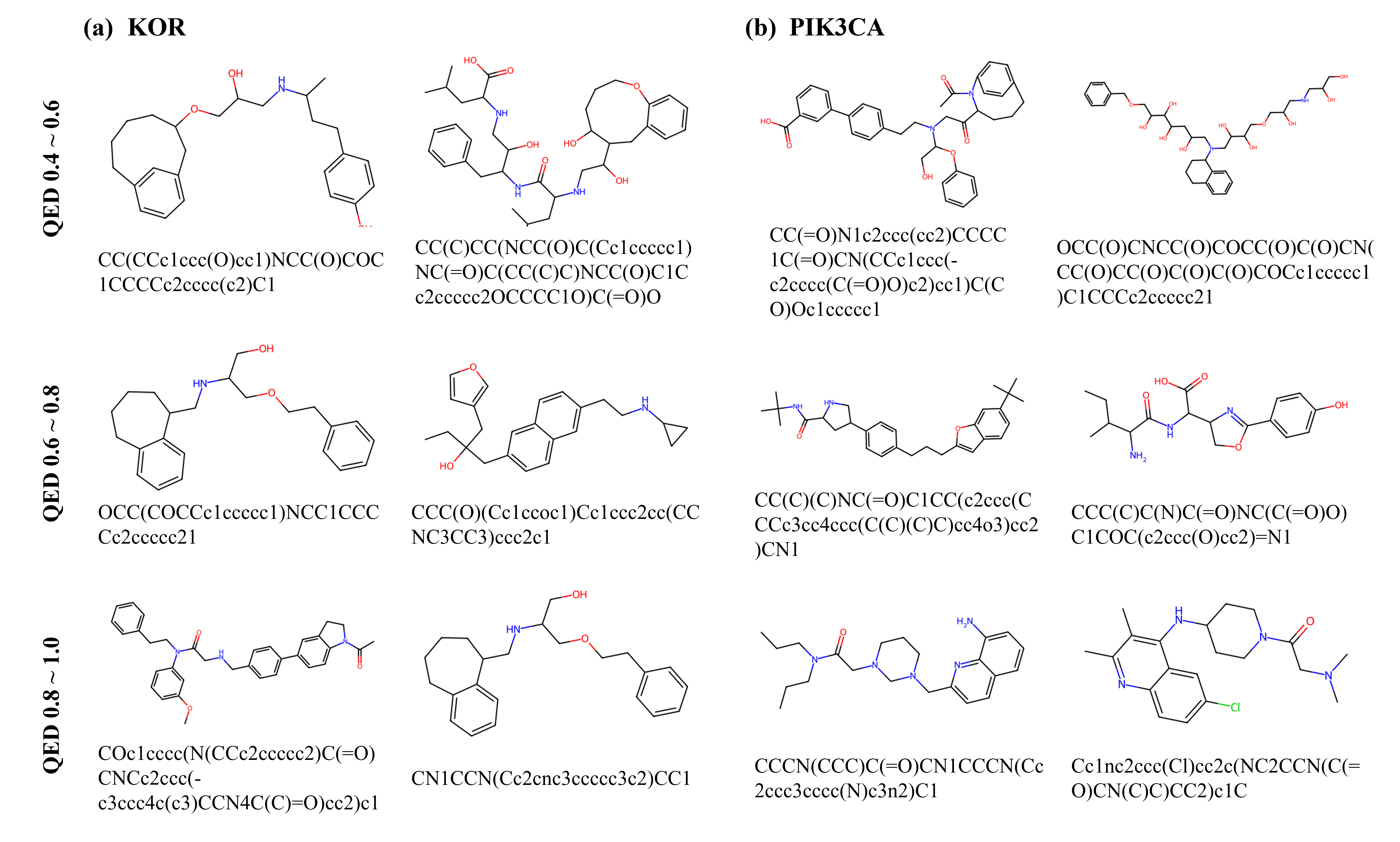

Supplement: Supplementary file 8 — Additional file 8 [file 13321_2026_1221_MOESM8_ESM.png]

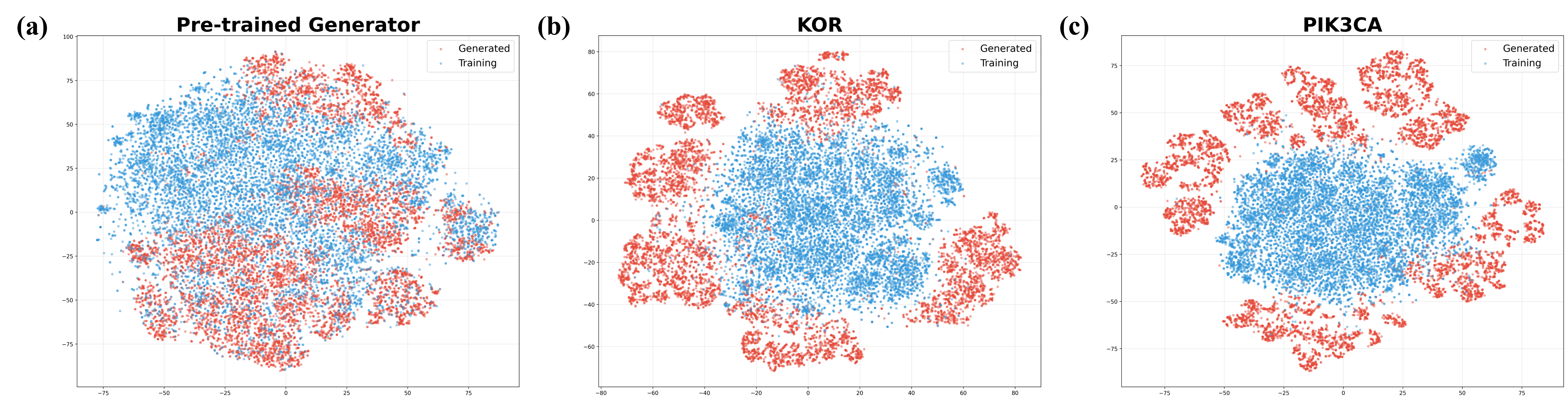

Supplement: Supplementary file 9 — Additional file 9 [file 13321_2026_1221_MOESM9_ESM.png]

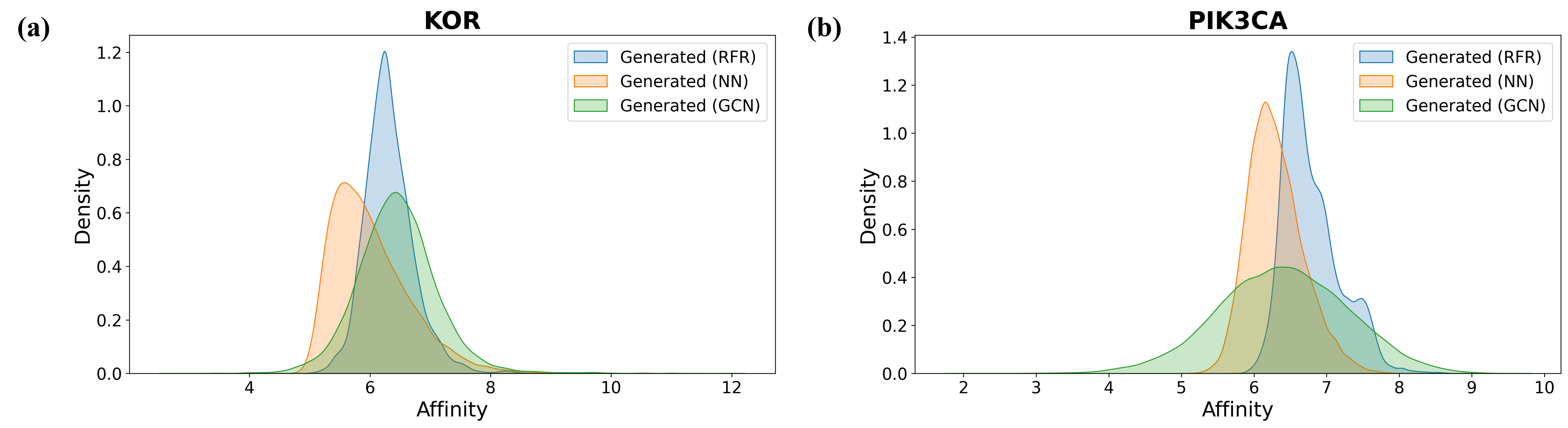

Supplement: Supplementary file 10 — Additional file 10 [file 13321_2026_1221_MOESM10_ESM.png]

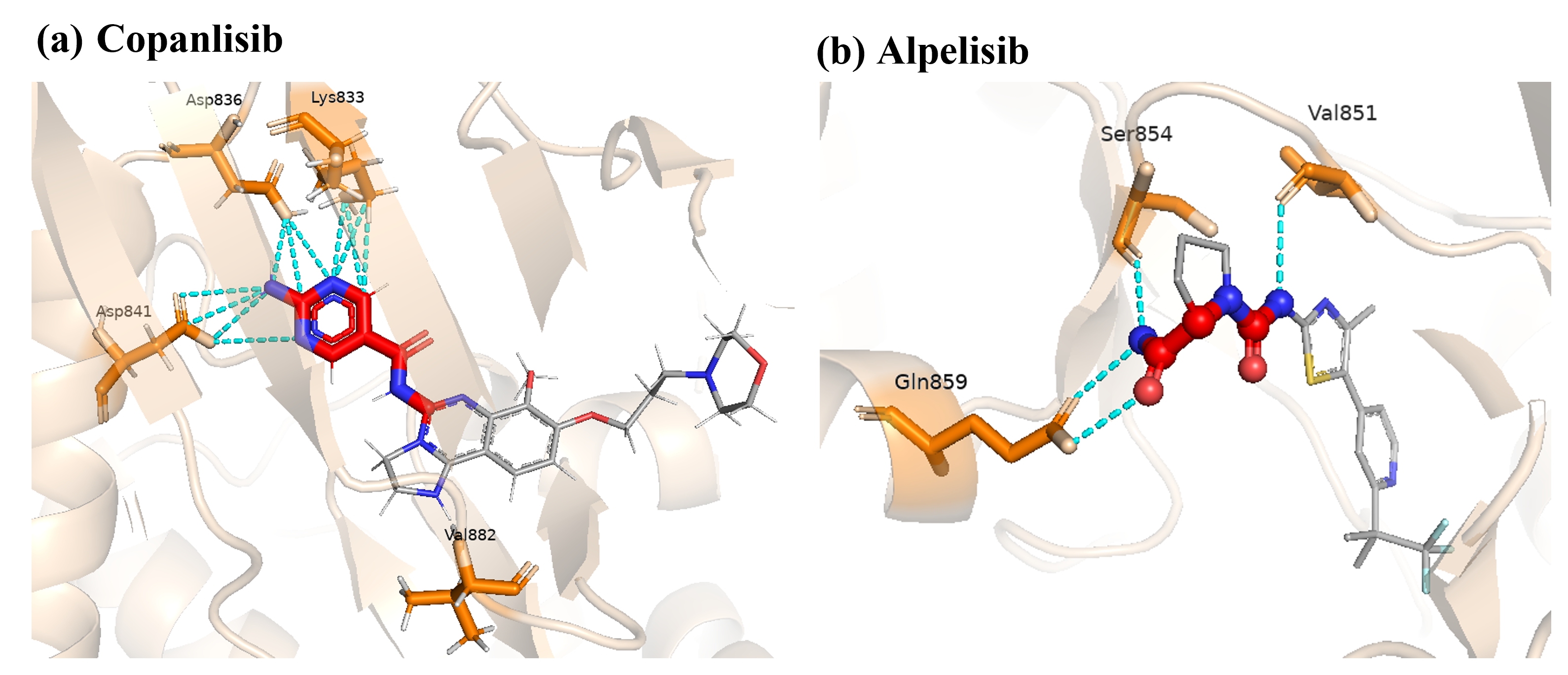

Supplement: Supplementary file 11 — Additional file 11 [file 13321_2026_1221_MOESM11_ESM.png]
