# Supplementary material for: Novel molecular design via a scaffold-aware transformer with multi-scale attention mechanisms
Source: J Cheminform. 2026 May 19;18:105. doi: 10.1186/s13321-026-01221-6 (PMC13425956; doi:10.1186/s13321-026-01221-6)
Supplement: Supplementary file 12 — Additional file 12 [file 13321_2026_1221_MOESM12_ESM.docx]

Supplementary Material

**Novel Molecular Design via a Scaffold-Aware Transformer with Multi-Scale Attention Mechanisms**

Junyoung Park ^a,b^, Sunyong Yoo ^a,b*^

^a^ Department of Intelligent Electronics and Computer Engineering, Chonnam National University, Gwangju, Republic of Korea

^b^ R&D Center, MATILO AI Inc., Gwangju, Republic of Korea

*** Corresponding author. Chonnam National University, 77 Yongbong-ro, Buk-gu, Gwangju, 61186, Korea, College of Engineering, Building 7, Republic of Korea**

**E-mail addresses:** [**sss206391@gmail.com**](mailto:sss206391@gmail.com) **(J. Park),** [**syyoo@jnu.ac.kr**](mailto:syyoo@jnu.ac.kr) **(S. Yoo)**

**Section 1. Model hyperparameters and supervised fine-tuning settings**

This section summarizes the hyperparameter configurations and computational requirements used for training and fine-tuning the proposed framework. We conducted all experiments on an NVIDIA A100-PCIE-40GB GPU with 40 GB memory, using CUDA 11.2, Python 3.7.16, and PyTorch 1.12.1. The peak GPU memory consumption was approximately 28 GB for generator pre-training, 1.3 GB for predictor training, and 40 GB for the full fine-tuning framework including both models, experience memory, and tournament selection. The generator was trained for 10 epochs over the GuacaMol dataset, requiring approximately 4 hours and 10 minutes of total training time. Fine-tuning was conducted for 30 epochs on each target dataset, requiring approximately 43 hours and 10 minutes per target. The generator hyperparameters are presented in Table S1. The generator was optimized using the AdamW optimizer with a weight decay of 0.1. The predictor hyperparameters are presented in Table S2. For each dataset, we performed hyperparameter optimization using Optuna, and conducted training for up to 1,000 epochs with early stopping based on validation loss to prevent overfitting. The fine-tuning configurations, including experience replay and layered tournament selection settings, are summarized in Table S3.

We designed the fine-tuning hyperparameters based on the interplay between experience memory dynamics and multi-objective selection. The per-epoch generation size was set equal to the memory capacity of 50,000 to ensure sufficient candidate volume for memory renewal at each iteration. We sample 20,000 molecules from the experience memory per epoch, maintaining a ratio of approximately 40% relative to new generations to balance exploitation and exploration. The three-stage tournament selection eliminates half of the candidates at each stage using a distinct criterion, resulting in a survival rate of one-eighth that reflects the three optimization objectives. We adopted a fixed budget of 30 epochs rather than early stopping because the tournament-based optimization does not produce a monotonic validation signal; the optimization curves in Section 7 and Fig. S6 empirically confirm convergence within this budget.

During fine-tuning, scaffold-conditioned generation was performed using a fixed set of five reference scaffolds. The scaffolds were configured to span chemically distinct frameworks, including heteroaromatic systems, benzofused heterocycles, and flexible diaryl and amine frameworks with varying heteroatom content. At each sampling step, the generator cycled through the five scaffolds in a batch-wise manner to ensure balanced presentation of all scaffolds during experience collection and subsequent generator updates. The five reference scaffolds are listed below as SMILES: (1) c1cnc2c(N3CCCNCC3)cccc2c1 (2) c1ccc(C2=NOCC2)cc1 (3) c1ccc(Cc2cc3c(CNC4CCCCC4)cccc3o2)cc1 (4) c1ccc(CCCNC2CCCCC2OCCCc2ccccc2)cc1 (5) O=C(CCN(Cc1ccccc1)c1cccc(-c2ccccc2)c1)N1CCN(c2ccccc2)CC1.

**Table S1.** Hyperparameters of the generator.

| **Hyper parameter** | **Setting** |
| --- | --- |
| Vocabulary size | 96 |
| Maximum sequence length | 102 |
| Transformer layers | 8 |
| Attention heads | 8 |
| Embedding dimension | 512 |
| Dropout | 0.1 |
| Scaffold weight | 2.0 |
| Learning rate | 6×10⁻⁴ |
| Loss function | Focal loss  (alpha=0.25, gamma=1) |

**Table S2.** Hyperparameters of predictor for the KOR and PIK3CA datasets.

| **Hyper parameter** | **KOR** | **PIK3CA** |
| --- | --- | --- |
| Node feature dimension | 9 | 9 |
| GATConv layers | 3 | 3 |
| Initial filters | 27 | 53 |
| Embedding dimension | 176 | 105 |
| Output dimension | 70 | 165 |
| Attention heads | 10 | 10 |
| Dropout | 0.2 | 0.3 |
| Learning rate | 3×10⁻⁴ | 3×10⁻⁴ |
| Loss function | MSE | MSE |

**Table S3.** Tournament selection and experience replay settings used during supervised fine-tuning.

| **Hyper parameter** | **Setting** |
| --- | --- |
| Epochs | 30 epochs |
| Batch size | 200 |
| Learning rate | 1×10⁻⁴ |
| Experience memory size | 50,000 Molecules |
| New generation per epoch | 50,000 SMILES |
| Memory sampling per epoch | 20,000 SMILES |
| Tournament layers | 3 sequential layers |
| Scores per layer | Layer 1: predicted activity  Layer 2: agent NLL  Layer 3: − prior NLL |

**Section 2. SMILES sequence generation process**

The SMILES sequence generation process is depicted in Fig. S1. Before generating the sequence, the generator requires predefined initial tokens and scaffold conditions. The generation process starts by providing the start token [SOS] and the desired scaffold condition as inputs to the generator. The generator predicts the next-token probability distribution by applying the softmax function to its output logits. According to this probability distribution, the next token is sampled and added to the current sequence. The extended sequence is then used again as an input to the generator to predict the subsequent token. This process repeats until the [EOS] token is generated or the length of the generated sequence reaches the predefined maximum length of 100 tokens. Once generation is complete, the generator returns a sequence of token indices. Using the predefined vocabulary, these indices are converted into their corresponding tokens, which are then concatenated to obtain the final SMILES string.


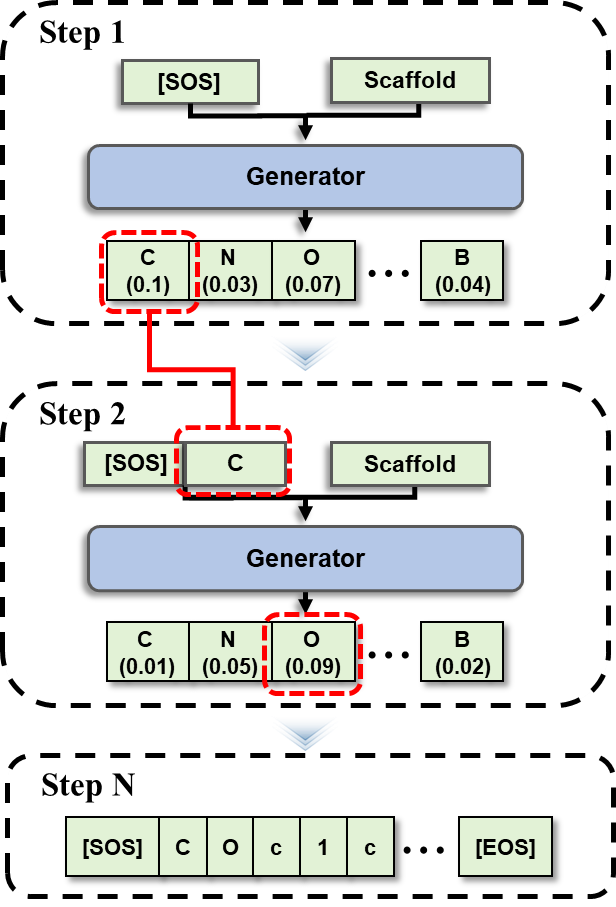


**Fig. S1.** The process of a new molecular generation. The step-by-step process to generate new molecules using the generator. In the first step, the next token (C) is predicted based on the [SOS] token and the input scaffold. The predicted token is concatenated with the [SOS] token. In the second step, the next token (O) is predicted based on the concatenated sequence and the scaffold. This process continues up to 100 iterations or until an [EOS] token is generated.

**Section 3. Ablation study to examine the impact of different components on the performance of the generative model**

We conducted an ablation study to examine the impact of different components on the performance of the generative model, comparing three variants: (i) generator trained using cross-entropy as the loss function, (ii) generator trained with multi-scale attention using scales 1 through 5, and (iii) generator trained without applying multi-scale attention. We compared these models to assess the impact of the loss function and multi-scale attention on the quality and diversity of the generated molecules. The results underscore the importance of multi-scale attention mechanisms and suitable loss functions in enhancing the capacity of the model to generate molecules with the desired properties. The performance differences between the proposed model and these ablated models are presented in Fig. S2. The generator using cross-entropy achieved a validity of 0.014 higher than the proposed model, but recorded a reduction of 0.17 in the uniqueness. The generator with multi-scale attention, using scales 1 through 5, achieved validity and uniqueness values that were 0.07 lower than those of the proposed model. Lastly, the generator without applying multi-scale attention recorded a validity that was 0.019 lower and a uniqueness that was 0.159 lower than the proposed model. All models achieved a novelty score of 1.0. While the model using cross-entropy achieved higher validity than the proposed model, its uniqueness value was markedly lower. The two remaining variants recorded lower values in both validity and uniqueness.

**
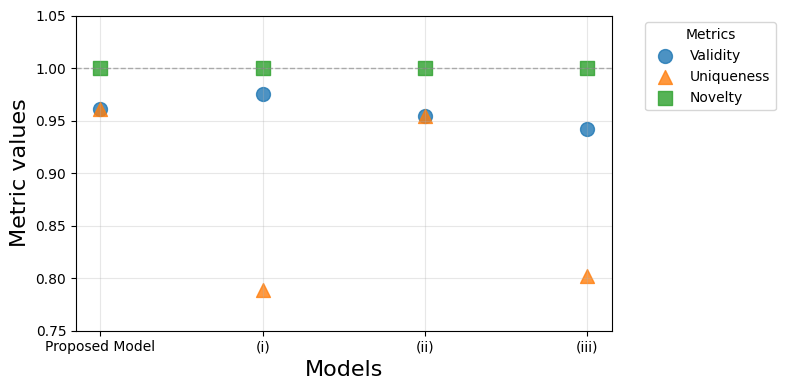
**

**Fig. S2.** Results of the ablation study on the proposed model. A comparison of the performance of three variants: (i) generator trained using cross-entropy as the loss function, (ii) generator trained with multi-scale attention using scales 1 through 5, and (iii) generator trained without applying multi-scale attention. The x-axis presents each specific model, and the y-axis presents the metric values.

**Section 4. Comparison of token-wise probability distributions at different temperatures during SMILES generation**

Validity and uniqueness typically show a trade-off during SMILES generation. When validity decreases, the denominator in the calculation of uniqueness becomes smaller, leading to an increase in uniqueness. Temperature controls sampling randomness by rescaling the model output before the softmax function. When temperature is lower, the token probability distribution becomes sharper and sampling favors the highest probability tokens more strongly. When temperature is higher, the distribution becomes flatter and sampling explores lower probability tokens more frequently. For a fixed model output at a given generation step, lowering temperature increases the probability mass on higher probability tokens and decreases the probability mass on lower probability tokens. Fig. S3 illustrates this sharpening and flattening effect using the same conditioning input and the same generation step so that the probability profiles differ only by temperature scaling. We compared multiple temperature settings and selected 0.9 because it provided the most balanced validity and uniqueness performance in our experiments. The results are summarized in Table S4.


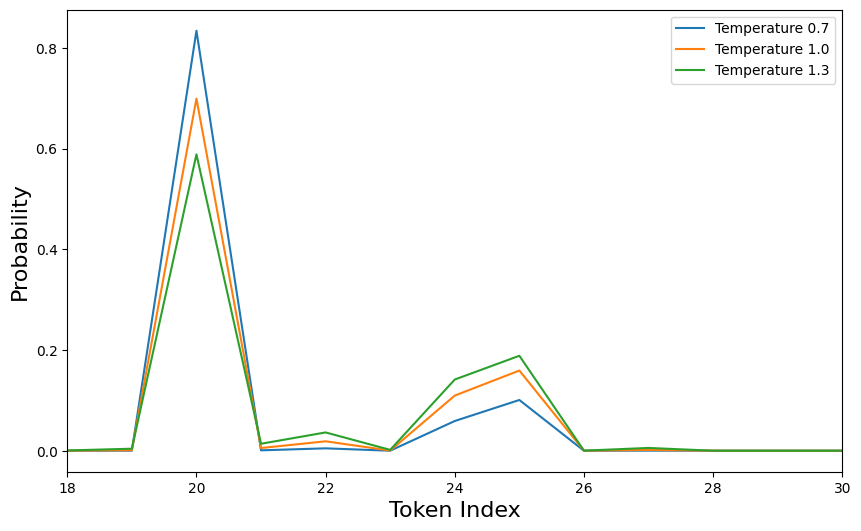


**Fig. S3.** Probability distributions over tokens at different temperature settings during SMILES generation. The x-axis presents the token index, and the y-axis presents the probability assigned to each token. The blue line represents the probability distribution at temperature 0.7, the red line represents the distribution at temperature 1.0, and the green line represents the distribution at temperature 1.3.

**Table S4.** The performance comparison of the generative model at different temperatures.

| **Temperature** | **Validity** | **Uniqueness** | **Novelty** |
| --- | --- | --- | --- |
| 0.7 | 0.982 | 0.875 | 1.0 |
| 0.8 | 0.968 | 0.912 | 1.0 |
| 0.9 | 0.961 | 0.961 | 1.0 |
| 1.0 | 0.942 | 0.971 | 1.0 |

**Section 5. Learning dynamics of scaffold awareness**

We monitored the scaffold retention rate throughout the pre-training process. Scaffold retention was defined as the proportion of valid generated molecules that contain the conditioning scaffold as a substructure. At each epoch, 1,000 molecules were generated for each of three representative scaffolds with varying complexity: benzene (simple), diphenylmethane (medium), and benzamide (complex). Substructure matching was performed using RDKit. Fig. S4 illustrates the learning dynamics of the generator. The scaffold retention rate increased progressively from approximately 86% at epoch 1 to over 98% by epoch 8, demonstrating that scaffold awareness emerges early in training and strengthens as the model converges. Notably, the retention rate showed accelerated improvement between epochs 2 and 6, coinciding with the steepest decrease in validation loss. After epoch 8, both metrics stabilized, indicating that the model had learned to reliably incorporate scaffold constraints into its generation process. These results confirm that the multi-scale scaffold attention mechanism effectively guides the model to preserve the conditioning scaffold throughout training.


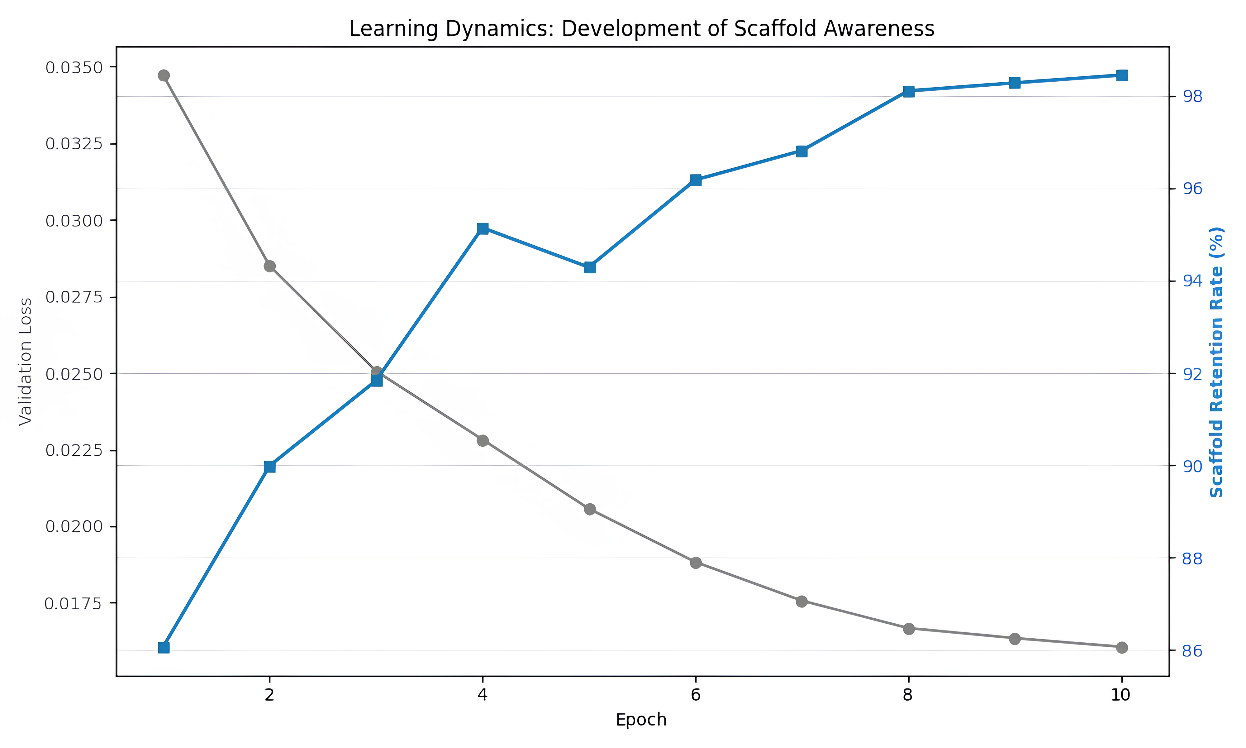


**Fig. S4.** Learning dynamics of the generator during pre-training. The gray line represents validation loss (left axis), and the blue line represents scaffold retention rate (right axis). Scaffold awareness develops progressively throughout training, with the retention rate improving from 86.1% to 98.2% over 10 epochs.

**Section 6. Generalization and robustness validation**

We evaluated the generalization capability of the pre-trained generator on scaffolds that were not present in the training dataset. Ten scaffolds were randomly selected from the held-out test set, ensuring they were completely absent from the training data. For each scaffold, 10,000 SMILES were generated, and this process was repeated five times with different random seeds. The results are reported as mean ± standard deviation. Table S5 presents the generation performance on unseen scaffolds. The pre-trained generator achieved an average validity of 0.958, uniqueness of 0.962, and novelty of 1.0 across all unseen scaffolds. These results are comparable to the performance observed on test set scaffolds (Table 2 in the main text), demonstrating that the model generalizes to novel scaffold structures rather than simply memorizing training set decorations. The consistent performance across both seen and unseen scaffolds confirms that the multi-scale scaffold attention mechanism effectively learns generalizable patterns for scaffold-conditioned molecular generation. The two-dimensional chemical structures of the scaffolds used in Table 2 and Table S5 are illustrated in Fig. S5.

**Table S5.** Performance evaluation of the pre-trained generator on scaffolds not seen during training.

| **Scaffold** | **Validity** | **Uniqueness** | **Novelty** | |
| --- | --- | --- | --- | --- |
| O=S(=O)(OCc1ccccc1)c1ccccc1 | 0.966 ± 0.003 | 0.936 ± 0.002 | 1.000 ± 0.000 |  |
| O=C(NCc1ccccc1)C(Cc1ccccc1)Nc1ccnc(NCCc2cccnc2)n1 | 0.939 ± 0.002 | 0.995 ± 0.002 | 1.000 ± 0.000 | |
| c1ccc(OCCCc2cnc3ccccc3c2)cc1 | 0.975 ± 0.001 | 0.959 ± 0.003 | 1.000 ± 0.000 | |
| O=S1(=O)C2CCNCCC2CN1CCc1ccccc1 | 0.940 ± 0.002 | 0.990 ± 0.001 | 1.000 ± 0.000 | |
| c1ccc(CCCCn2ccc3ncccc32)cc1 | 0.955 ± 0.002 | 0.929 ± 0.004 | 1.000 ± 0.000 | |
| S=C(C[n+]1ccccc1)SCc1ccccc1 | 0.944 ± 0.002 | 0.965 ± 0.002 | 1.000 ± 0.000 | |
| O=C(CN1CCCNCC1)NC1CCCC1 | 0.980 ± 0.002 | 0.950 ± 0.003 | 1.000 ± 0.000 | |
| O=C(COc1ccccc1)NC1CC2CCC(C1)N2 | 0.970 ± 0.001 | 0.981 ± 0.001 | 1.000 ± 0.000 | |
| O=C(CCC1CCCC1)NCCCN1CCOCC1 | 0.962 ± 0.003 | 0.960 ± 0.002 | 1.000 ± 0.000 | |
| c1nc2c(c(-c3ccsc3)n1)CCNCC2 | 0.956 ± 0.003 | 0.962 ± 0.002 | 1.000 ± 0.000 | |
| Average | 0.958 | 0.962 | 1.0 | |


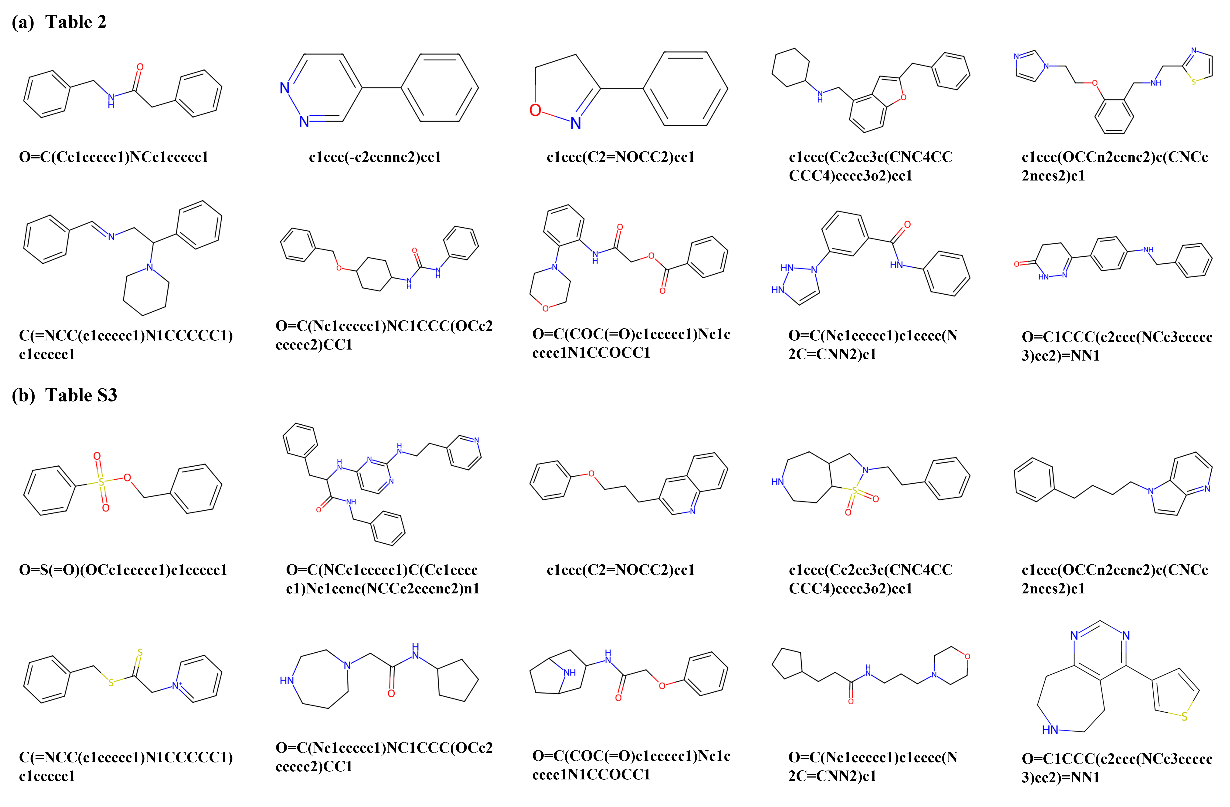


**Fig. S5.** Two-dimensional chemical structures of the scaffolds used for generator evaluation. (a) Scaffolds from the test set used in Table 2 and 5. (b) Scaffolds completely absent from the training dataset used in Table S5.

We performed 5-fold cross-validation to assess the robustness of the GAT-based predictor across different train/test splits. The dataset was partitioned into five folds, and for each fold, the model was trained on four folds and evaluated on the held-out fold. Within each training partition, 18.75% of the data was reserved for validation to select the optimal model checkpoint based on validation loss. All models were trained for 1,000 epochs, and the best-performing weights on the validation set were retained for testing. The cross-validation results are summarized in Table S6. The GAT predictor achieved a mean test MSE of 0.416 ± 0.054 and R² of 0.788 ± 0.028 for KOR, and a mean test MSE of 0.444 ± 0.061 and R² of 0.744 ± 0.027 for PIK3CA. The low standard deviations across folds demonstrate that the predictor performance is robust and not dependent on a specific data split.

**Table S6.** 5-fold cross-validation results of the GAT-based predictor.

| **Dataset** | **Test MSE** | **Test R²** |
| --- | --- | --- |
| KOR | 0.416 ± 0.054 | 0.788 ± 0.028 |
| PIK3CA | 0.444 ± 0.061 | 0.744 ± 0.027 |

**Section 7. Optimization curves during supervised fine-tuning**

We conducted 30 epochs of fine-tuning on both the KOR and PIK3CA datasets and recorded performance metrics at each iteration. The tracked metrics include validity, uniqueness, novelty, and predicted activity score (PredAct). As illustrated in Fig. S6, the PredAct score exhibits a consistent upward trend throughout the fine-tuning process for both targets. This monotonic improvement reflects the direct selection pressure imposed by our tournament-based optimization strategy, which explicitly selects molecules with higher predicted activity scores for subsequent training epochs. For the KOR target, PredAct increased from approximately 6.2 to 6.5, while for the PIK3CA target, it improved from approximately 7.4 to 8.0 over 30 epochs. In contrast, the distributional metrics (validity, uniqueness, and novelty) exhibit epoch-to-epoch fluctuations rather than monotonic trends. This behavior is expected and can be attributed to three factors: (1) inherent sampling noise during molecular generation, (2) the fact that these metrics are not directly incorporated into the optimization objective, and (3) the exploration–exploitation trade-off inherent to the fine-tuning process. Notably, despite these fluctuations, the variation magnitudes remain relatively small—validity consistently exceeds 0.96, uniqueness remains above 0.98, and novelty stays near 1.0 throughout training. This demonstrates that the fine-tuning procedure effectively improves the targeted property while maintaining stable molecular quality and diversity.


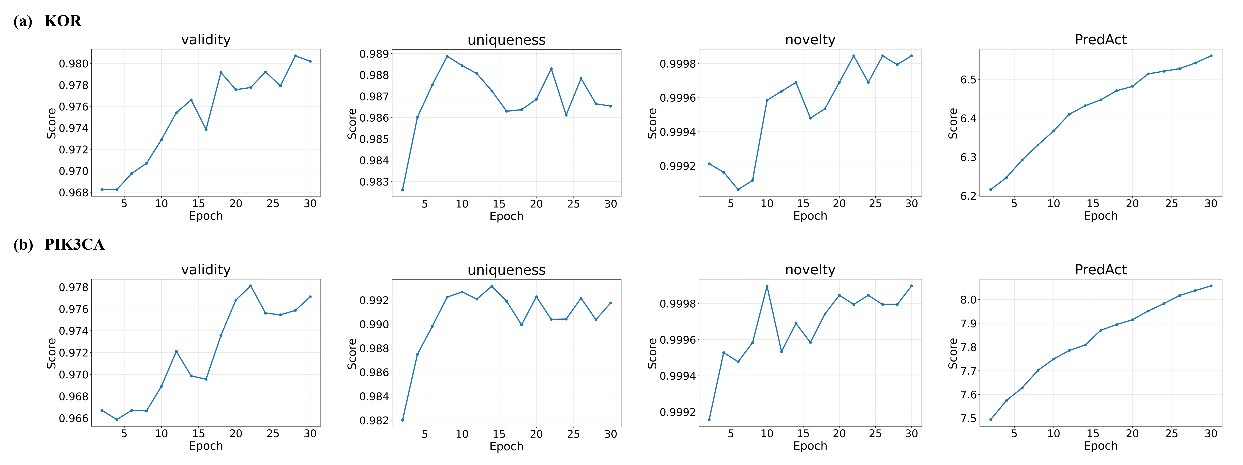


**Fig. S6.** Optimization curves during supervised fine-tuning for (a) KOR and (b) PIK3CA targets. Four metrics are tracked across 30 fine-tuning epochs: validity, uniqueness, novelty, and predicted activity score. The PredAct score shows consistent improvement due to direct optimization pressure, while other distributional metrics exhibit minor fluctuations within acceptable ranges.

**Section 8. Chemical properties of the generated molecules**

This study calculated the quantitative estimate of drug-likeness (QED) and the synthetic accessibility score (SAS) to evaluate the chemical properties of the generated molecules. The QED evaluates the likelihood that a molecule is a potential drug candidate on a scale of 0 to 1, whereas the SAS evaluates the synthetic feasibility of a molecule on a scale of 1 to 10. Higher QED values indicate greater drug-likeness, suggesting that these molecules are more promising as drug candidates. Lower SAS values indicate that the molecules can be synthesized with relative ease. We subsequently generated 15,000 molecules using the fine-tuned model and calculated the QED and SAS for each one. The distributions of the QED and SAS for the generated molecules are presented in Fig. S7. For the KOR, the QED values ranged from a minimum of 0.016 to a maximum of 0.947. Around 20% of all molecules had a QED score greater than 0.6, and around 7% had a QED score greater than 0.8. The SAS values ranged from a minimum of 1.83 to a maximum of 5.91. Around 99% of all molecules had an SAS of less than 5, and approximately 32% had an SAS of less than 3. For PIK3CA, the QED values ranged from a minimum of 0.028 to a maximum of 0.947. Around 46% of all molecules had a QED greater than 0.6, and around 11% had a QED greater than 0.8. The SAS values ranged from a minimum of 1.66 to a maximum of 5.04. Approximately 99% of all molecules had an SAS of less than 5, and 60% had an SAS of less than 3. To provide a visual representation of the molecular quality across different QED ranges, we selected representative molecules from three QED bins: 0.4 to 0.6, 0.6 to 0.8, and 0.8 to 1.0. Fig. S8 illustrates example molecules from each QED bin for both KOR and PIK3CA targets. Molecules with higher QED scores generally exhibit more balanced molecular properties, including appropriate molecular weight, lipophilicity, and favorable structural features for drug-likeness.


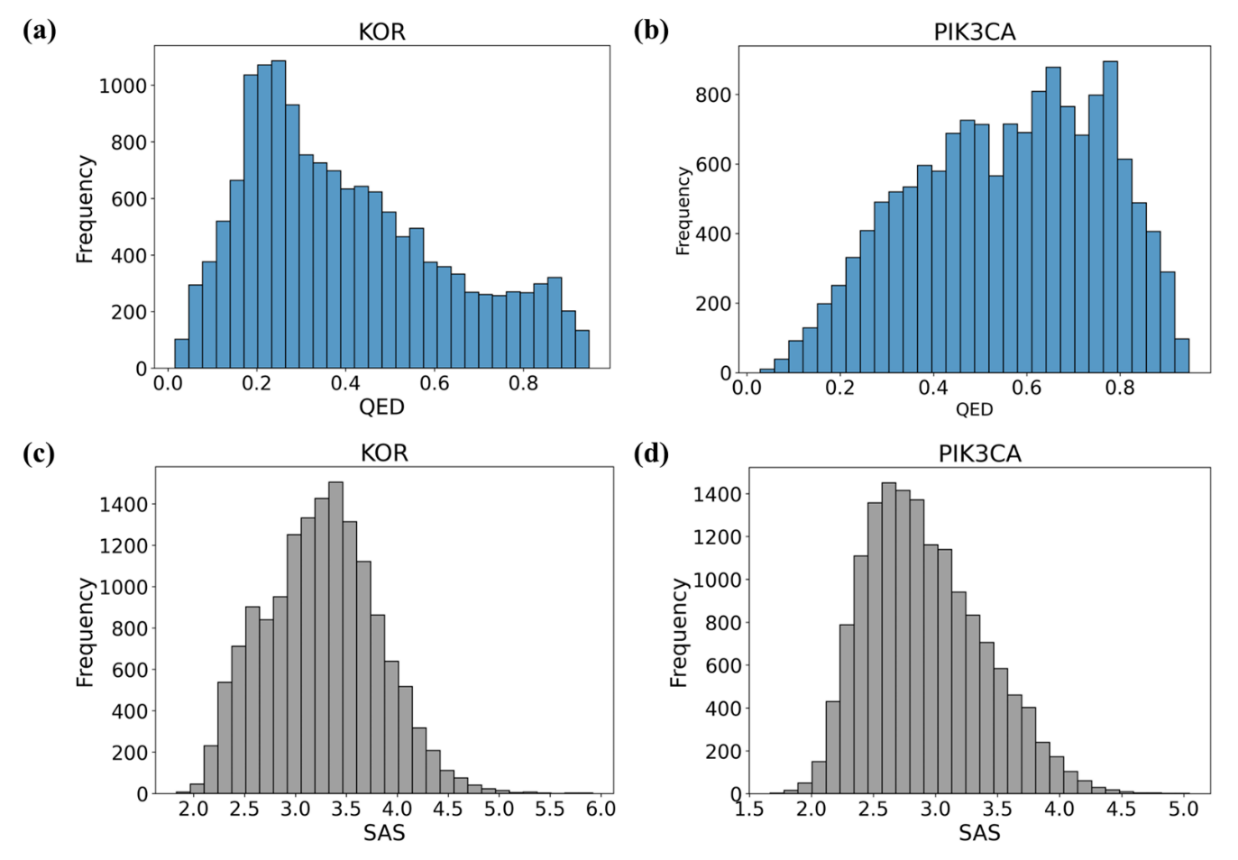


**Fig. S7.** Distributions of the QED and SAS of the generated molecules. (a, b) QED distributions for the KOR and PIK3CA datasets. (c, d) SAS distributions for the KOR and PIK3CA datasets. The x-axis presents the QED or SAS, and the y-axis presents the frequency of molecules.


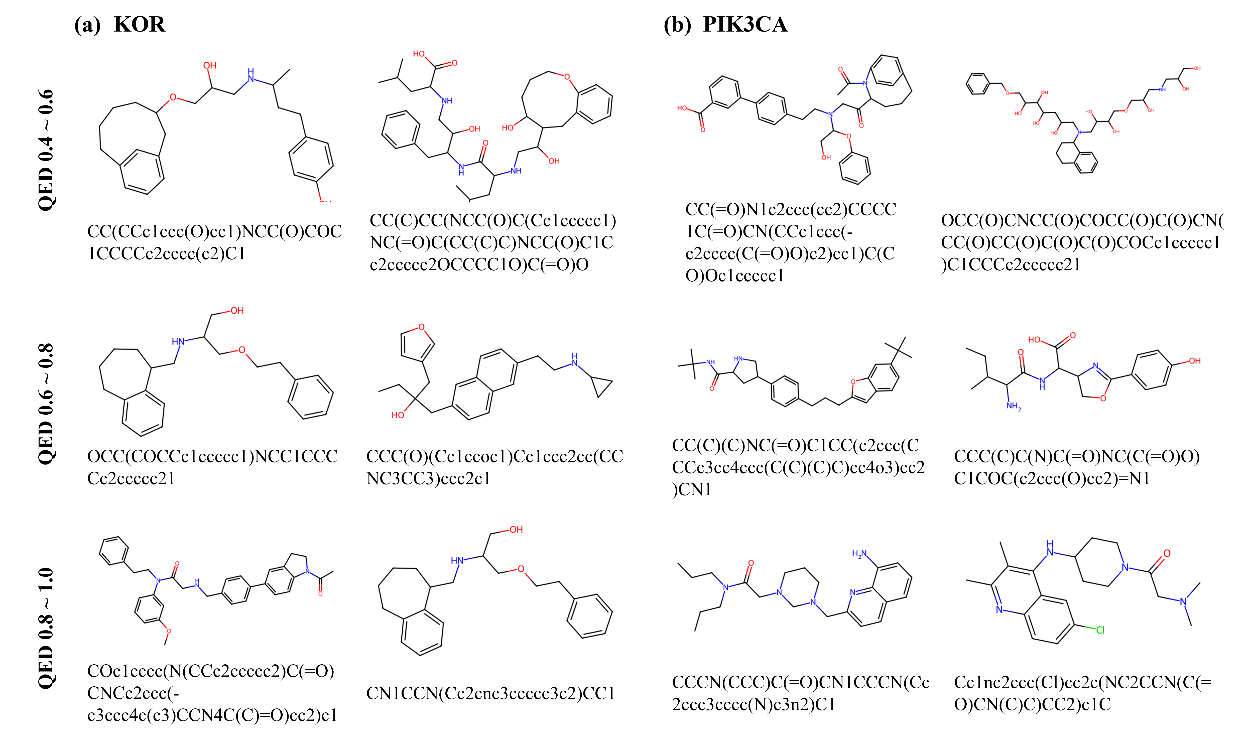


**Fig. S8.** Representative molecules from different QED score ranges generated by the fine-tuned model. (a) KOR target and (b) PIK3CA target. Each row corresponds to a QED bin: 0.4 to 0.6, 0.6 to 0.8, and 0.8 to 1.0. Molecules with higher QED scores exhibit more favorable drug-like properties.

**Section 9. Chemical space and distance analysis**

We performed dimensionality reduction analysis using t-SNE to evaluate the chemical space coverage of the generated molecules. We randomly sampled 10,000 valid molecules from each generated set and 10,000 valid molecules from the training dataset. Morgan fingerprints with a radius of 2 and 2048 bits were computed for all molecules. Principal component analysis was first applied to reduce the fingerprint dimensionality to 50 components, and t-SNE was subsequently applied with perplexity of 30 and learning rate of 200 to project the representations into a two-dimensional space. Fig. S9 presents the t-SNE visualizations comparing the chemical space distributions across three model configurations. The pre-trained generator produces molecules that substantially overlap with the training data distribution, confirming that the model successfully learned the chemical space of drug-like molecules. For the fine-tuned models targeting KOR and PIK3CA, the generated molecules maintain overlap with the training distribution while extending into peripheral regions. This pattern indicates that the fine-tuning process guides the model toward target-specific chemical space while preserving the capacity to explore structurally novel compounds. The distinct clustering patterns observed in the fine-tuned models suggest that the optimization process effectively shifts the molecular distribution toward regions associated with predicted biological activity.

We quantified the distance between generated molecules and training data using two metrics: nearest-neighbor Tanimoto similarity and Fréchet ChemNet Distance (FCD). For each generated molecule, the maximum Tanimoto similarity to any molecule in the training set was computed using Morgan fingerprints with radius 2 and 2048 bits. The nearest-neighbor distance was then calculated as one minus this maximum similarity. FCD measures the distributional difference between two molecular sets in a learned chemical feature space. A total of 5,000 valid molecules were randomly sampled from each set for this analysis. Table S7 presents the distance analysis results. The pre-trained generator showed a mean nearest-neighbor Tanimoto similarity of 0.316 and FCD of 9.57, indicating that generated molecules explore novel chemical space while maintaining reasonable proximity to known drug-like structures. The fine-tuned models for KOR and PIK3CA exhibited slightly lower similarities of 0.306 and 0.297, respectively, with higher FCD values of 16.64 and 17.61. This divergence from the training distribution reflects the target-specific optimization process, where the models successfully shifted toward distinctive regions of chemical space associated with higher predicted activities.


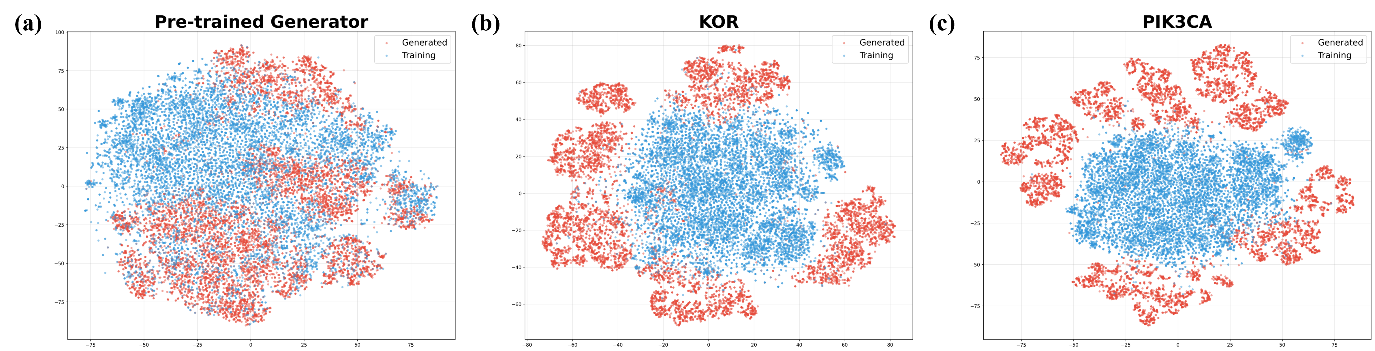


**Fig. S9.** t-SNE visualization of chemical space comparing generated molecules and training data. Red points represent generated molecules and blue points represent training data molecules. The three panels show comparisons for (a) the pre-trained generator, (b) the KOR fine-tuned model, and (c) the PIK3CA fine-tuned model.

**Table S7.** Distance analysis between generated molecules and training data.

| **Model** | **Mean 1-NN Similarity** | **Mean 1-NN Distance** | **FCD** |
| --- | --- | --- | --- |
| Pre-trained Generator | 0.316 | 0.684 | 9.57 |
| KOR | 0.306 | 0.694 | 16.64 |
| PIK3CA | 0.297 | 0.703 | 17.61 |

**Section 10. External validation using alternative prediction models**

External validation was performed to ensure that the predicted bioactivity of generated molecules is not biased by the specific architecture of our GAT-based predictor. Three alternative prediction models were independently trained on the same bioassay datasets: Random Forest Regressor with Morgan fingerprints as input features, a feed-forward neural network with Morgan fingerprints, and a graph convolutional network operating directly on molecular graphs. These alternative predictors were trained on the same training split and evaluated on an identical held-out test set to ensure fair comparison. The performance metrics of all predictors on the test set are summarized in Table S8. All models achieved R-squared values above 0.65 for both KOR and PIK3CA datasets, demonstrating that the activity signals are consistently captured regardless of the prediction architecture. The GAT-based predictor used in our framework achieved comparable or superior performance, supporting the reliability of the predicted bioactivity values reported in the main text. The predicted bioactivity distributions of the fine-tuned generated molecules by each alternative predictor are shown in Fig. S10. For the KOR dataset, all three independent predictors estimated mean activities ranging from approximately 5.5 to 7.0, which is consistent with the GAT-predicted mean activity of 6.56 reported in Table 3. For the PIK3CA dataset, the independent predictors estimated mean activities in the range of 6.0 to 7.5. The GAT-predicted mean activity of 8.05 is slightly higher than these estimates, which can be attributed to optimization bias during supervised fine-tuning where the GAT predictor was used as the reward signal. Nevertheless, all independent predictors consistently classified the generated molecules within the active range, confirming that the high predicted activities are not artifacts of the GAT architecture but reflect genuine structural features learned by the generator.

**Table S8.** External validation results using different activity prediction models.

| **Dataset** | **Model** | **MSE** | **R²** |
| --- | --- | --- | --- |
| KOR | GAT (ours) | 0.416 | 0.788 |
|  | Random forest | 0.524 | 0.706 |
|  | Neural Network | 0.507 | 0.716 |
|  | GCN | 0.616 | 0.655 |
| PIK3CA | GAT (ours) | 0.444 | 0.744 |
|  | Random forest | 0.524 | 0.732 |
|  | Neural Network | 0.504 | 0.739 |
|  | GCN | 0.623 | 0.681 |


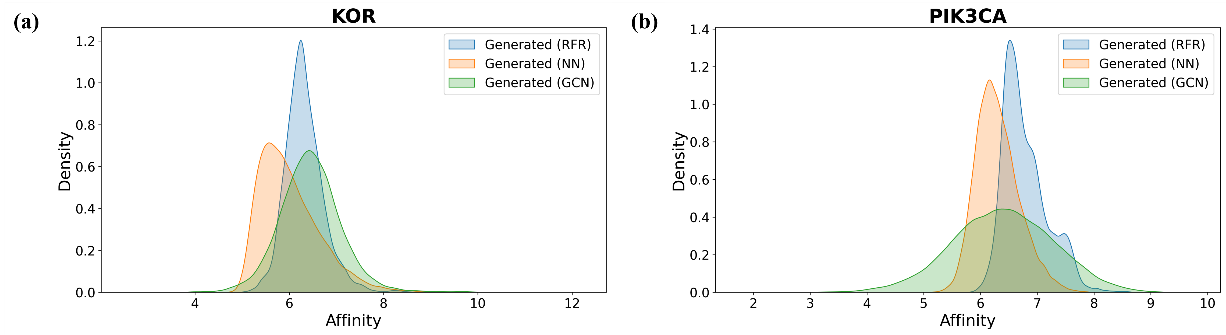


**Fig. S10.** Predicted bioactivity distributions of fine-tuned generated molecules evaluated by independent prediction models. (a) KOR dataset and (b) PIK3CA dataset. Three alternative predictors were used: Random Forest Regressor (RFR), feed-forward neural network (NN), and graph convolutional network (GCN). All models were trained independently from the GAT-based predictor used in our framework.

**Section 11. Scaffold retention analysis of baseline models**

We analyzed scaffold retention for PromptSMILES and LibINVENT by generating 10,000 valid molecules per scaffold across 10 representative scaffolds. Four retention metrics were computed: exact Murcko scaffold match, Murcko similarity at threshold 0.6, analog count at threshold 0.6, and mean Tanimoto similarity between generated and input scaffolds. Table S9 and Table S10 summarize the scaffold retention results for PromptSMILES and LibINVENT respectively.

PromptSMILES achieved an exact match of 1.0 for 9 out of 10 scaffolds and maintained high similarity mean values above 0.9 for scaffolds with complex multi-ring systems such as c1ccc(Cc2cc3c(CNC4CCCCC4)cccc3o2)cc1 and O=C(COC(=O)c1ccccc1)Nc1ccccc1N1CCOCC1. However, simple scaffolds such as c1ccc(-c2ccnnc2)cc1 showed substantially lower Murcko similarity of 0.031 and analog fraction of 0.001, indicating that while the scaffold substructure was preserved, the generated molecules contained significant additional structural modifications that altered the Murcko framework. LibINVENT demonstrated exact scaffold retention of 1.0 for 9 out of 10 scaffolds, with one scaffold, O=C(COC(=O)c1ccccc1)Nc1ccccc1N1CCOCC1, showing 0.0 exact retention. This failure occurred because the LibINVENT attachment point decomposition for this scaffold produced R-groups that altered the Murcko framework upon reassembly. The overall Murcko similarity values for LibINVENT were generally lower than PromptSMILES for simple scaffolds but higher for complex scaffolds, with a mean similarity range of 0.464 to 0.840. Compared to our Scaffold-Aware Transformer, which achieved variable exact retention ranging from less than 1% to 86.5% with a mean of 38.7%, both baselines showed higher exact retention rates due to their explicit scaffold-conditioning mechanisms. However, this higher retention came at the cost of reduced chemical diversity and lower predicted bioactivity as demonstrated in Table 3 and Table 4 of the main manuscript.

**Table S9.** Scaffold retention analysis of PromptSMILES.

| **Scaffold** | **Exact** | **Similarity**  **(τ =0.6)** | **Analog**  **(τ =0.6)** | **Similarity Mean** |
| --- | --- | --- | --- | --- |
| O=C(Cc1ccccc1)NCc1ccccc1 | 1.000 | 0.713 | 0.025 | 0.853 |
| c1ccc(-c2ccnnc2)cc1 | 1.000 | 0.031 | 0.001 | 0.369 |
| c1ccc(C2=NOCC2)cc1 | 1.000 | 0.433 | 0.020 | 0.672 |
| c1ccc(Cc2cc3c(CNC4CCCCC4)cccc3o2)cc1 | 1.000 | 1.000 | 1.000 | 0.998 |
| c1ccc(OCCn2ccnc2)c(CNCc2nccs2)c1 | 1.000 | 1.000 | 1.000 | 1.000 |
| C(=NCC(c1ccccc1)N1CCCCC1)c1ccccc1 | 1.000 | 0.985 | 0.618 | 0.984 |
| O=C(Nc1ccccc1)NC1CCC(OCc2ccccc2)CC1 | 1.000 | 0.999 | 0.941 | 0.998 |
| O=C(COC(=O)c1ccccc1)Nc1ccccc1N1CCOCC1 | 1.000 | 1.000 | 1.000 | 1.000 |
| O=C(Nc1ccccc1)c1cccc(N2C=CNN2)c1 | 1.000 | 0.999 | 0.200 | 0.999 |
| O=C1CCC(c2ccc(NCc3ccccc3)cc2)=NN1 | 1.000 | 0.99800 | 0.000 | 0.999 |

**Table S10.** Scaffold retention analysis of LibINVENT.

| **Scaffold** | **Exact** | **Similarity**  **(τ =0.6)** | **Analog**  **(τ =0.6)** | **Similarity Mean** |
| --- | --- | --- | --- | --- |
| O=C(Cc1ccccc1)NCc1ccccc1 | 1.000 | 0.532 | 0.437 | 0.666 |
| c1ccc(-c2ccnnc2)cc1 | 1.000 | 0.234 | 0.080 | 0.508 |
| c1ccc(C2=NOCC2)cc1 | 1.000 | 0.224 | 0.000 | 0.464 |
| c1ccc(Cc2cc3c(CNC4CCCCC4)cccc3o2)cc1 | 1.000 | 0.954 | 0.927 | 0.807 |
| c1ccc(OCCn2ccnc2)c(CNCc2nccs2)c1 | 1.000 | 1.000 | 0.000 | 0.548 |
| C(=NCC(c1ccccc1)N1CCCCC1)c1ccccc1 | 1.000 | 0.909 | 0.816 | 0.840 |
| O=C(Nc1ccccc1)NC1CCC(OCc2ccccc2)CC1 | 1.000 | 0.953 | 0.925 | 0.838 |
| O=C(COC(=O)c1ccccc1)Nc1ccccc1N1CCOCC1 | 0.000 | 0.641 | 0.641 | 0.616 |
| O=C(Nc1ccccc1)c1cccc(N2C=CNN2)c1 | 1.000 | 0.802 | 0.695 | 0.765 |
| O=C1CCC(c2ccc(NCc3ccccc3)cc2)=NN1 | 1.000 | 0.879 | 0.828 | 0.801 |

**Section 12. Three-dimensional binding mode visualization for attention analysis**

We generated three-dimensional binding mode visualizations to validate whether the attention-highlighted substructures correspond to known protein-ligand interactions. Crystal structures of Copanlisib and Alpelisib bound to PI3Kα were obtained from the Protein Data Bank and visualized using PyMOL. The attention-highlighted atoms were displayed as red spheres, and key interacting residues were labeled to enable direct comparison with the two-dimensional attention maps presented in Fig. 5.

Fig. S11a shows the binding mode of Copanlisib within the PI3Kα active site. The attention-highlighted aminopyrimidine region forms direct hydrogen bonds with Asp836, Asp841, and Lys833 in the affinity pocket, consistent with the crystallographic binding model reported in previous studies. Fig. S11b shows the binding mode of Alpelisib, where the attention-highlighted proline-derived carboxamide and 2-aminothiazole region interacts with Gln859, Ser854, and Val851.


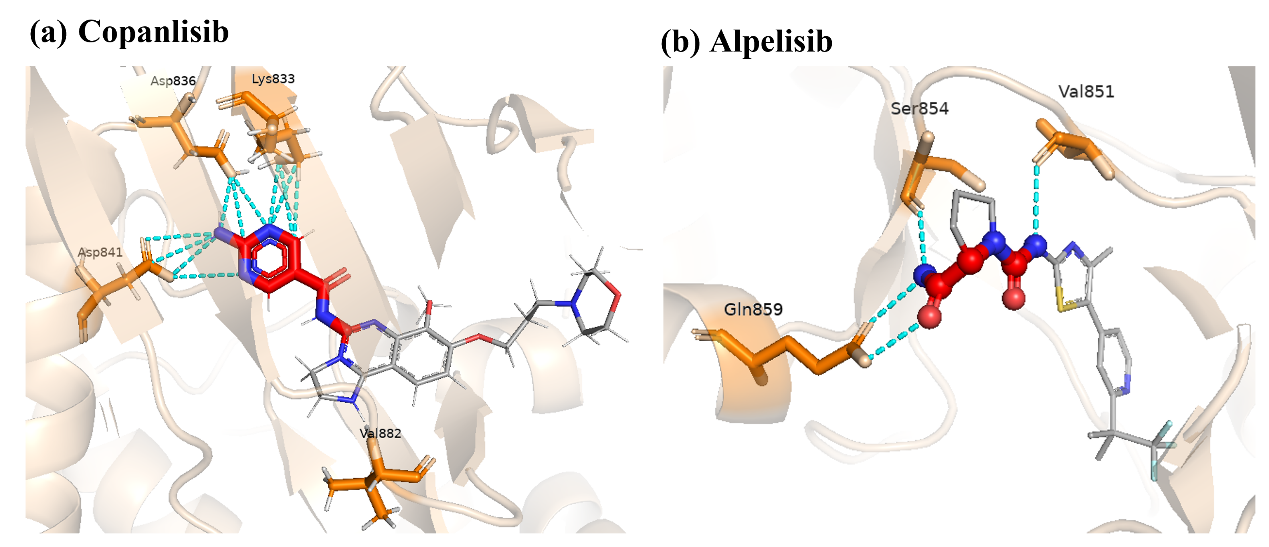


**Fig. S11.** Three-dimensional binding modes of PIK3CA inhibitors with attention-highlighted substructures. Attention-highlighted atoms are shown as red spheres, and key interacting residues are displayed as orange sticks. Hydrogen bonds are indicated by cyan dashed lines. (a) Copanlisib bound to PI3Kα, showing the aminopyrimidine group forming hydrogen bonds with Asp836, Asp841, and Lys833 in the affinity pocket. (b) Alpelisib bound to PI3Kα, showing the proline-derived carboxamide and 2-aminothiazole region interacting with Gln859, Ser854, and Val851.

**Section 13. SAR transfer analysis across different scaffolds**

We investigated whether the structure-activity relationship knowledge learned during fine-tuning can transfer to scaffolds not seen during training. Five held-out scaffolds were selected from the MOSES dataset, ensuring that they were not present in the fine-tuning data for either target. For each scaffold, 10,000 molecules were generated using three generator configurations: the pre-trained baseline generator, the KOR fine-tuned generator, and the PIK3CA fine-tuned generator. All generated molecules were scored using the same PIK3CA predictor to evaluate whether KOR-specific fine-tuning could improve predicted activities on a different target.

Table S11 presents the SAR transfer analysis results. Across all five held-out scaffolds, the KOR fine-tuned generator consistently produced molecules with higher mean predicted activities compared to the baseline generator, despite being optimized for a different target. The mean activity scores increased by 0.01 to 0.29 units compared to baseline across scaffolds. The PIK3CA fine-tuned generator achieved the highest scores as expected, since it was directly optimized for the scoring predictor. These results suggest that fine-tuning captures generalizable SAR patterns that can partially transfer across different molecular targets, enabling the generation of higher-activity molecules even for scaffolds and targets not encountered during optimization.

However, this analysis has limitations. The evaluation relies on in silico predictions from a computational model rather than experimental binding assays or molecular docking simulations. Additionally, the degree of SAR transfer may depend on the structural similarity between the training and test scaffolds, as well as the relatedness of the target proteins. Further experimental validation would be required to confirm these findings in a wet-lab setting.

**Table S11.** SAR transfer analysis results across five held-out scaffolds.

| **Test scaffold** | **Pre-trained model** | | **KOR-tuned model** | | | **PIK3CA-tuned model** | | |
| --- | --- | --- | --- | --- | --- | --- | --- | --- |
|  | **Mean** | **Top 5%** | | **Mean** | **Top 5%** | | **Mean** | **Top 5%** |
| Oc1c(Br)cc(Cl)c2cccnc12 | 7.115 | 8.396 | | 7.330 | 8.736 | | 7.512 | 8.754 |
| Cc1oncc1C(=O)Nc1ccc(C(F)(F)F)cc1 | 7.327 | 8.510 | | 7.344 | 8.530 | | 7.451 | 8.565 |
| COc1ccccc1OC(=O)c1ccccc1OC(C)=O | 7.621 | 8.466 | | 7.743 | 8.646 | | 7.826 | 8.684 |
| O=C1CC(c2ccccc2)Nc2nc3ccccc3n21 | 7.205 | 8.465 | | 7.491 | 8.637 | | 7.687 | 8.803 |
| Cc1ccnc(NC(=O)c2ccc(C(=O)Nc3cc(C)ccn3)cc2)c1 | 7.549 | 8.685 | | 7.615 | 8.640 | | 7.742 | 8.729 |

**Section 14. Molecular quality filter analysis**

We evaluated the chemical quality of generated molecules using LillyMedChem-style filters implemented through RDKit FilterCatalog. The filter pipeline includes PAINS (A, B, and C), BRENK, NIH, and ZINC alert catalogs, which identify molecules containing known reactive, promiscuous, or undesirable substructural motifs. For each model, the same 20,000 generated SMILES used in Table 3 and Table 4 were evaluated. The pass rate is defined as the fraction of RDKit-valid molecules that contain no flagged substructures.

Table S12 summarizes the LillyMedChem filter pass rates for all models across both targets. PromptSMILES achieved the highest pass rates of 0.8157 for KOR and 0.8394 for PIK3CA, followed by LOGICS at 0.5158 and 0.6057 respectively. Our Scaffold-Aware Transformer showed the lowest pass rates at 0.3686 for KOR and 0.2923 for PIK3CA.

The inverse relationship between predicted bioactivity and filter pass rate across models is consistent with the known trade-off between potency optimization and chemical quality in generative molecular design. Our model, which achieved the highest PredAct values across both targets, generates molecules that are more aggressively optimized toward the bioactivity objective, resulting in a higher proportion of molecules containing substructural alerts. PromptSMILES, which achieved the lowest PredAct for PIK3CA, produces molecules closer to the general drug-like chemical space of its prior, leading to higher filter pass rates.

This trade-off can be addressed by incorporating chemical quality filters as additional scoring components during fine-tuning. The modular design of our tournament-based SFT framework readily supports the integration of filter-based penalties without modifying the generator architecture, which represents a straightforward direction for future optimization.

**Table S12.** LillyMedChem filter pass rates for generated molecules.

| **Models** | | **Filter rate** |
| --- | --- | --- |
| KOR | Scaffold-aware transformer | 0.3686 ± 0.001 |
|  | LOGICS | 0.5158 ± 0.002 |
|  | PromptSMILES | 0.8157 ± 0.001 |
|  | LibINVENT | 0.4773 ± 0.001 |
| PIK3CA | Scaffold-aware transformer | 0.2923 ± 0.008 |
|  | LOGICS | 0.6057 ± 0.009 |
|  | PromptSMILES | 0.8394 ± 0.007 |
|  | LibINVENT | 0.4688 ± 0.001 |
